# Carbohydrate metabolism and autophagy signature predicts prognosis and immune microenvironment of acute myeloid leukemia

**DOI:** 10.7717/peerj.21168

**Published:** 2026-05-22

**Authors:** Qingchun Shen, Lan Xiao, Jing Liu, Miao Zhang, Jiabo Ding, Ling Guo, Qulian Guo, Jing Guo, Tingting Leng, Wenjun Liu, You Yang

**Affiliations:** 1 Institute of Animal Sciences, Chinese Academy of Agricultural Sciences, Beijing, China; 2 Southwest Medical University, Luzhou, Sichuan, China

**Keywords:** Acute myeloid leukemia, Carbohydrate metabolism, Autophagy, Prognosis, Immune microenvironment

## Abstract

The existing risk stratification for acute myeloid leukemia (AML) reveals considerable heterogeneity in patient prognosis, underscoring the necessity for innovative risk stratification methodologies to optimize treatment responses. In this multicohort study, we explored the potential of carbohydrate metabolism and autophagy-related genes (CARGs) to enhance prognostic classification in AML patients. Employing univariate regression and least absolute shrinkage and selection operator (LASSO)-Cox stepwise regression analysis, we constructed a prognostic signature involving four genes related to CARGs in AML patients. By leveraging data from the TCGA cohort with 117 patients, the Gene Expression Omnibus (GEO) public data cohort with 1,431 patients, and our internal cohort of 117 patients, we showcased the robustness and accuracy of the CARG signature in forecasting survival outcomes among a collective sample of 1,665 non-Acute Promyelocytic Leukemia (APL) patients. Patients were categorized into high-risk and low-risk groups based on median risk score. The overall survival (OS) was significantly shorter in the high-risk group compared to the low-risk group. Differentially expressed genes (DEGs) were identified. Gene Ontology (GO) and Gene Set Enrichment Analysis (GSEA) analysis revealed that the DEGs were primarily associated with immune response signaling pathways. Immune-related analysis indicated that patients classified in the high-risk group exhibited a suppressive immune microenvironment. The results of the potential drugs for the risk groups demonstrated that inhibitors of PI3K/AKT/mTOR signaling pathway were effective. The novel risk model based on CARGs proposed in our study shows promise in prognostic classifications in AML, potentially providing new insights for the development of precise targeted cancer therapies.

## Introduction

Acute myeloid leukemia (AML) is marked by considerable genetic, epigenetic, and clinical heterogeneity (Vago & Gojo, 2020; Arber et al., 2022; Döhner et al., 2022). Standard curative approaches for AML typically involve chemotherapy, either as a standalone treatment or in conjunction with allogeneic stem cell transplantation (Döhner et al., 2017). The long-term survival probability for AML patients is approximately 35% to 40% for those under the age of 60, while it diminishes to between 5% and 15% for patients over 60 (Döhner, Weisdorf & Bloomfield, 2015). A significant challenge arises from the fact that a subset of AML patients classified as low risk by the European Leukemia Network (ELN) may still encounter poor prognoses in clinical settings. This underscores the urgent need for more precise stratification methods for AML to improve patient outcomes.

Carbohydrate metabolism comprises intricate pathways such as glycolysis, oxidative phosphorylation (OXPHOS), the hexosamine biosynthetic pathway (HBP), the pentose phosphate pathway (PPP), and and glycolipid synthesis, among others (Chandel, 2021). These interconnected biochemical processes play a crucial role in maintaining the balance of carbohydrate metabolism within cells, providing essential energy and vital precursors for biosynthesis. Autophagy, a key component of cellular defense mechanisms in tumors, significantly contributes to the degradation of damaged organelles and dysfunctional proteins, as well as the recycling of metabolites, thereby maintaining the stability of the internal environment (White, 2012).

Carbohydrate metabolism not only supported the survival of cancer cells but also influenced anti-tumor immunity and resistance by intricately regulating autophagy in cancer (Brohée et al., 2018; Kim et al., 2018; Li et al., 2018; La Belle Flynn et al., 2019; Chu et al., 2020; Lee et al., 2020; Hou et al., 2021; Marcucci & Rumio, 2022). Conversely, studies indicated that autophagy facilitated tumor progression, invasion, metastasis, and resistance through its regulation of carbohydrate metabolism (Fan et al., 2018; Jiao et al., 2018; Chien et al., 2022; Leonel et al., 2023; Zhou et al., 2023). The above evidence underscores the intricate interplay between autophagy and carbohydrate metabolism in tumor biology. It was observed that elevated autophagy correlated with increased respiration, enhanced mitochondrial ATP production, and greater glycolytic activity in AML (Gschwind et al., 2022). Further investigations have revealed that autophagy limited proliferation and glycolytic metabolism in acute myeloid leukemia (Watson et al., 2015). Additionally, researchers have discovered that autophagy was involved in lipid catabolism to support OXPHOS, and that inhibition of OXPHOS led to a reduction in autophagic flux in AML (Bosc et al., 2020).

While previous studies have established that carbohydrate metabolism and autophagy mutually regulate each other in AML, playing crucial roles in its pathophysiology, there remains a gap in the literature regarding the combined effects of these factors on prognosis, mutations, drug response, and immune response in AML. Therefore, developing a risk stratification model for AML that integrates carbohydrate metabolism and autophagy may provide valuable insights and promising avenues for enhancing patient prognosis and treatment strategies in this disease.

## Materials and Methods

### Process of the study

**Figure 1:**
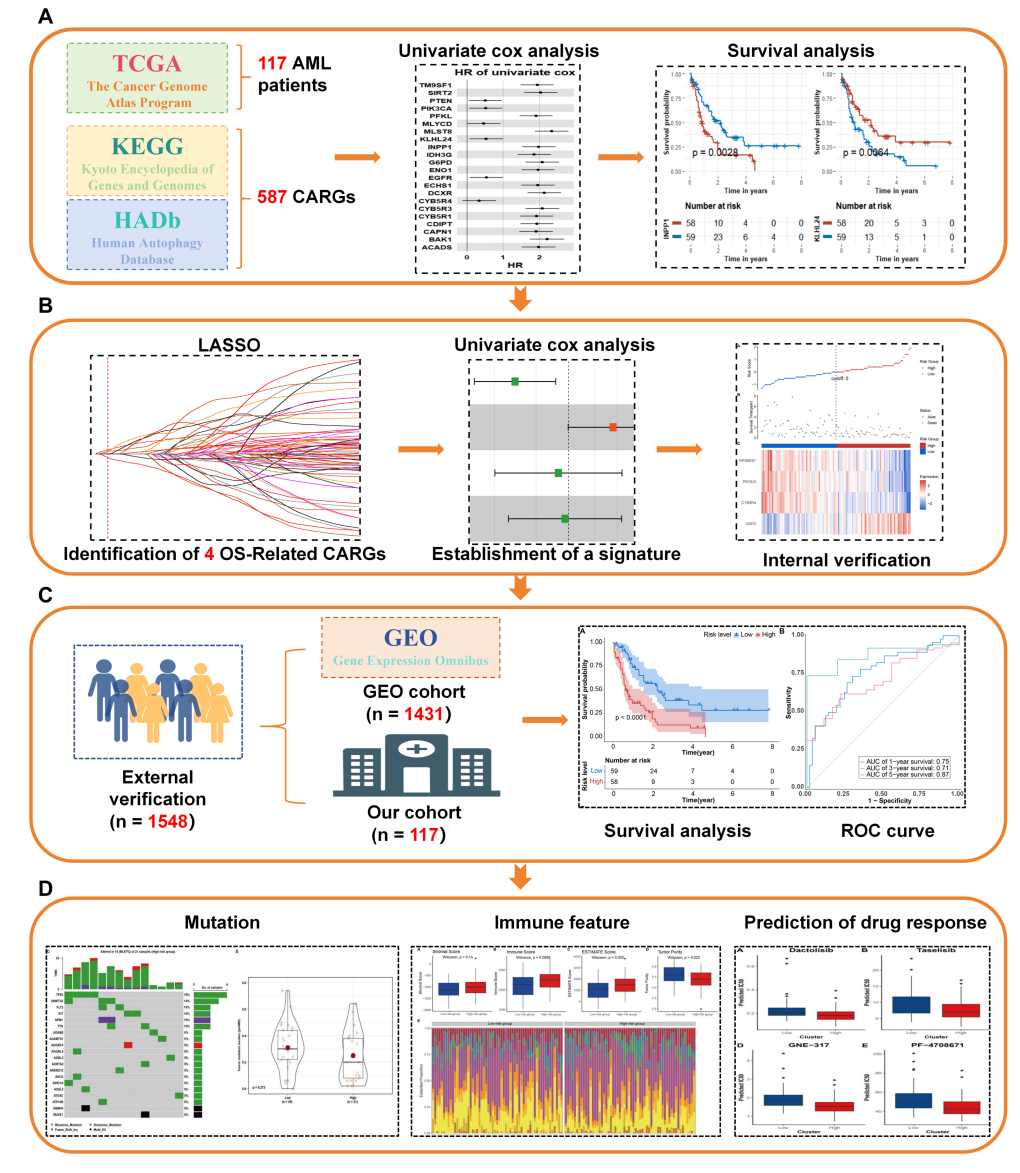
(A) Identification of OS-related CARGs in AML. (B) Establishment a strong CARG signature for prognostic assessment with combination of methods. (C) Validation of the prognostic significance of CARG signature in external cohorts. (D) Clinical characteristics and application of the CARG signature. LASSO, least absolute shrinkage and selection operator.

In this study, we systematically analyzed the impact of the combination of carbohydrate metabolism and autophagy-associated genes (CARGs) on prognosis, genetic mutations, immune response, and drug response in a sample of 1,665 AML patients. To provide a clear overview of our research methodology and findings, we have presented a flowchart (Fig. 1) that outlines the key steps and processes of our study. We utilized the AML training cohort (*n* = 117) from the Cancer Genome Atlas (TCGA) database and conducted the least absolute shrinkage and selection operator (LASSO) Cox analysis (Tibshirani, 1996). Through this analysis, we constructed a CARG signature that is associated with prognosis in AML. Internal validation was performed using bootstrap resampling (1,000 iterations) to estimate optimism-corrected performance metrics. The C-index and time-dependent AUCs were computed using the rms and timeROC packages, with 95% cofidence intervals estimated from the bootstrap distribution. To mitigate potential optimism, we performed the following: (1) Cross-validation was implemented during the LASSO tuning to prevent overfitting (Tibshirani, 1996). (2) The final model was validated on an independent dataset (bootstrap), which confirmed its stability and predictive performance. (3) We also evaluated feature stability across repeated resampling runs, and key predictors remained consistently selected.

We validated its prognostic value internally using the TCGA training set and further conducted external validation in the GEO testing cohort (*n* = 1,431) as well as in our own cohort (*n* = 117). Utilizing the CARG signature, we investigated the characteristics of mutations and immune cell profiles through the CIBERSORT (Newman et al., 2015) and Maftools (Mayakonda et al., 2018) packages. Additionally, we assessed the capacity of the CARG signature to predict patient responses to chemotherapeutic agents using the OncoPredict (Maeser, Gruener & Huang, 2021).

### Publicly available datasets and preprocessing

The RNA sequencing profiles, single nucleotide polymorphism (SNP) profiles, and comprehensive clinical data for AML datasets were obtained from public databases. Additionally, raw microarray datasets from GSE12417 (GPL96, *n* = 104), GSE37642 (GPL570, *n* = 243), GSE37642 (GPL96, *n* = 218), GSE71014 (GPL10558, *n* = 162), GSE106291 (GPL18460, *n* = 356), and GSE146173 (GPL18460, *n* = 348) datasets were sourced from the GEO (https://www.ncbi.nlm.nih.gov/geo/) and standardized using the normalizeBetweenArrays function within the limma package (Ritchie et al., 2015) for consistency across arrays. The AML RNA-seq dataset was acquired from TCGA database, which can be accessed at https://www.cancer.gov/about-nci/organization/ccg/research/structural-genomics/tcga. The TCGA database contained data on 151 AML patients. M3-AML, a subtype of AML that is well-characterized with extensive research on its etiology, molecular mechanisms, and treatment, was deliberately omitted from the clinical specimens in this study. Non-Acute Promyelocytic Leukemia (APL) patients were ultimately selected for further analysis, resulting in the enrollment of 1548 (GEO + TCGA) AML patients (Fig. 1). The available clinical information for these AML patients is detailed in Tables S1–S7.

### Our cohort

Patients with AML were diagnosed using the WHO 2008 criteria and categorized according to the FAB classification. The treatment approach for AML patients at the time of admission primarily followed the Chinese guidelines for the diagnosis and management of adult AML (non-APL) (2017) (Leukemia & Lymphoma Group, Chinese Society of Hematology, Chinese Medical Association, 2017). The ELN Criteria were utilized for prognostication of AML patients. In summary, 117 newly diagnosed AML patients (excluding the M3 subtype) were ultimately chosen at the Affiliated Hospital of Southwest Medical University between January 2019 and November 2024. Comprehensive clinical data was collected after the written consent of the patient, and the study, approved by the Affiliated Hospital of Southwest Medical University, was carried out in compliance with the principles of the Declaration of Helsinki (No. KY2022293). The clinical information and significant gene alterations of AML samples in our cohort were documented in Table S8.

### Identification of OS-related CARGs

A sum of 572 CARGs were extracted from the Kyoto Encyclopedia of Genes and Genomes (KEGG) pathway database (https://www.genome.jp/kegg/pathway.html#global) and the Human Autophagy Database (HADb) (https://www.autophagy.lu/v1/clustering/index.html). They are listed in Table S9. To investigate the potential prognostic significance of these CARGs in patients with AML, the TCGA cohort (*n* = 117) was utilized as a training set. The study aimed to identify CARGs associated with OS using univariate Cox proportional hazards regression analysis, with a significance threshold of *P* < 0.05.

### Development and validation of CARG prognostic signature for AML patients

Based Cox regression, the study employed the LASSO (Fontanarosa & Dai, 2011) method to identify the most significant features among the OS-related CARGs. Subsequently, a multivariate Cox proportional hazards regression was performed on these selected candidates using stepwise variable selection based on the Akaike information criterion (Vrieze, 2012). The risk score for the final prognostic features was calculated using the following formula:

\begin{eqnarray*}\text{Risk score}=\sum _{i}^{n}Coefi\times {A}_{i} \end{eqnarray*}

where Coef is the regression coefficient for the individual CARG in the CARG signature, “i” represents the CARG that composed of the CARG signature, “A” represents the relative expression value of the individual CARG, and “n” represents the number of genes in the signature. Following the calculation of the risk score, patients were stratified into high-risk and low-risk groups using the median risk score as the cutoff value. To guarantee identical model structure across platforms, the same linear predictor (coefficients derived from TCGA training set) was applied to the standardized expression matrix of each validation dataset. Differences in the OS of patients were then assessed using Kaplan–Meier analysis and the log-rank test. These statistical methods were employed to evaluate any significant disparities in the OS between the high-risk and low-risk groups. The predictive capacity of the CARG signature was evaluated using the time-dependent receiver operating characteristic (ROC) curve (Heagerty, Lumley & Pepe, 2000). To provide a more rigorous assessment of model generalizability, we reported optimism-corrected performance metrics (1,000 bootstrap resamples) and C-index with confidence intervals alongside time-dependent AUC (Steyerberg, 2016).

The predictive accuracy of the CARG signature was assessed in 1548 samples, including datasets from GEO (GSE12417, GSE37642, GSE71014, GSE106291, GSE146173, *n* = 1,431) and our institutional cohort (*n* = 117). The risk score for each patient was computed based on the CARG signature, and a Kaplan–Meier curve was utilized to illustrate its survival performance.

### Identification and enrichment analysis of DEGs

The DEseq2 package was utilized for the identification of DEGs between the high- and low-risk groups (Love, Huber & Anders, 2014). Subsequently, a heatmap representing the DEGs was generated using the ‘pheatmap’ package (Tian et al., 2021). The clusterProfiler package was employed to conduct Gene Ontology (GO) functional enrichment analysis and Gene Set Enrichment Analysis (GSEA), enhancing the comprehension of the functional roles of the DEGs in AML (Subramanian et al., 2005; Yu et al., 2012).

### Development of a CARG clinicopathologic nomogram

A nomogram combining the CARG signature with clinicopathologic parameters was constructed using the rms package to predict OS for each AML patient in the training set (Zhang & Kattan, 2017). The calibration curve was used to evaluate the predictive discrimination of the CARG signature for AML patients (Alba et al., 2017).

### AML-immune microenvironment landscape and potential implications characterized by the CARG signature

After extracting the transcriptome data, we calculated the immune purity and immune infiltration in the training set. This was based on the ImmuneScore, StromalScore, and ESTIMATEScore of the expression matrix using the “estimate” R package (Yoshihara et al., 2013). The distribution of 22 immune cell types, in each of the 117 AML samples, along with their immune infiltration scores, was determined through the utilization of the CIBERSORT algorithm (Newman et al., 2015). This data was then graphically represented using bar charts. Samples with a significance level of *P* < 0.05 were selected for subsequent investigation. The proportions were then contrasted between tumor tissues categorized into low- or high-risk groups using the Wilcoxon rank sum test. Additionally, Pearson’s correlation was examined between these proportions. The outcomes were visually illustrated through histograms, boxplots, and heatmaps, constructed using the “ggplot”, “corrplot”, and “corrplot” R packages, respectively.

### Prediction of clinical chemotherapeutic response

We used the OncoPredict R package (v1.3) to predict drug response, analyzing associations between risk groups and sensitivity to standard chemotherapy/targeted therapies (Maeser, Gruener & Huang, 2021). The Genomics of Drug Sensitivity in Cancer (GDSC2) dataset (October 2023 release) served as the training set, providing drug response (log(IC50)) and gene expression data for ∼200–300 compounds across ∼900 cancer cell lines. Results were visualized *via* ggplot2-generated boxplots. All GDSC2 panel drugs were analyzed by default; a complete drug list (names, targets, and screening data) is provided in Table S10.

### Gene expression levels acquired through quantitative real-time PCR

The RZ solution (Transgen, China) was utilized to extract total RNA from peripheral blood mononuclear cells (PBMCs) obtained from 117 newly diagnosed AML patients at our hospital. cDNA was synthesized employing the TranScript All-in-One First-Strand cDNA Synthesis SuperMix for qPCR kit (Transgen, China). Subsequently, the mRNA levels were assessed using the TranStart Tip Green Qpcr SuperMix (Transgen, China). The amplification process occurred as follows: 94 °C for 30 s in 1 cycle; followed by 94 °C for 5 s and 60 °C for 20 s, repeated for 40 cycles. The primer sequences can be found in Table S11. The 2−ΔΔCT method was utilized to determine the relative mRNA expression levels.

### Statistical analysis

Statistical analyses were conducted using R software version 4.2.0 (https://www.r-project.org/). OS was estimated using the Kaplan–Meier method, and log-rank tests were conducted. Statistical differences between risk groups were evaluated using Chi-square (X2), Mann–Whitney, or Fisher’s exact tests as appropriate. For comparing two specified groups, Student’s *t*-test (two-tailed, unpaired) was applied with significance levels denoted as follows: *p < 0.05; **p < 0.01; ***p < 0.001; and ns for not significant.

## Results

### Identification of survival-related CARGs in AML

As the training set, we acquired gene expression profiles from 117 non-APL samples in the TCGA dataset. Subsequently, a combined total of 587 CARGs were obtained from both the KEGG dataset (355 CARGs) and the Human Autophagy Database (232 CARGs). Utilizing univariate Cox proportional hazards regression analysis, we investigated the potential prognostic value of each CARG. From the analysis, we identified a subset of 70 genes that exhibited significant associations with OS (Fig. S1A). Out of the 70 genes, 28 genes (Table S12) were found to be protective factors, while the remaining 42 genes were risk factors (Table S13). Furthermore, survival analysis demonstrated a significant correlation between high expression of the 28 protective factors and improved prognosis (Fig. S2), whereas high expression of the 42 risk factors was significantly associated with a worse prognosis in AML (Fig. S3). This validation supports the findings of the univariate Cox analysis.

### Development and validation of CARG signature

To mitigate the risk of overfitting, we employed the LASSO regression algorithm with minimum lambda to further screen and identify four key CARGs associated with OS from the initial pool of 70 CARGs (Figs. S1A and S1B). Followed by multivariate Cox proportional hazards regression analysis, the regression coefficients for these four CARGs were obtained and employed to establish an optimal CARG signature for patients’ OS (Fig. S1 C). Utilizing the expression levels and regression coefficients, a patient’s risk score was determined as follows: Risk score = [Expression level of CYB5R4 * (−0.36826)] + [Expression level of G6PD * (0.30701)] + [Expression level of PIK3CA * (−0.06915)] + [Expression level of RPS6KB1 * (−0.02365)].

**Figure 2:**
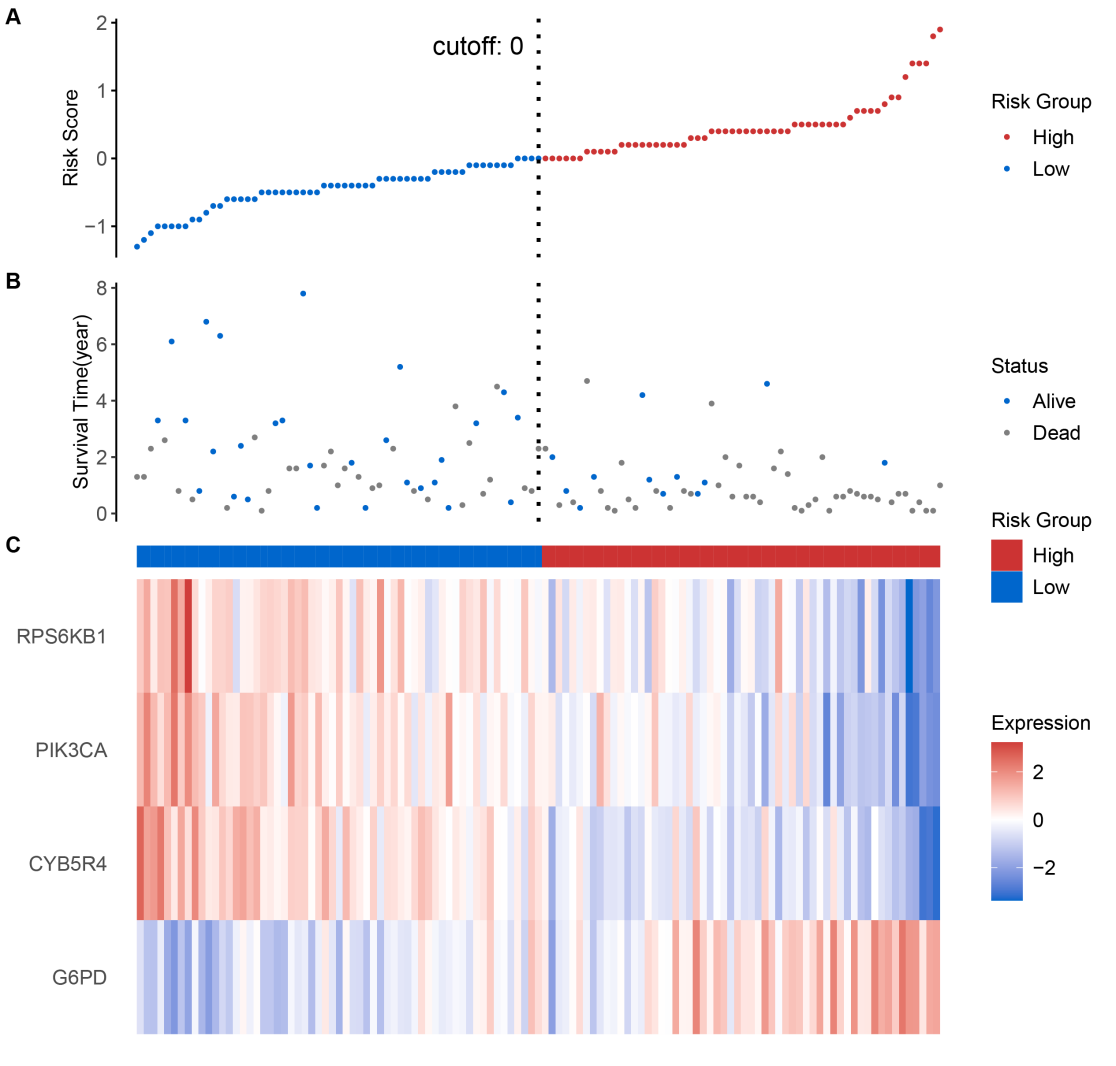
(A) Risk stratification based on patients’ risk scores. (B) Patients’ survival status correlated with their risk scores. (C) The expressions of the 4 model CARGs in the high- and low-risk groups. CARGs with *P* values less than below 0.05 were selected.

Based on the median risk score, patients were categorized into low-risk and high-risk groups. Upon the analysis, we observed a correlation between escalating risk scores and an increase in the number of deaths, particularly noting a higher mortality rate in the high-risk group as opposed to the low-risk group in training set (Figs. 2A, 2B). In terms of the expressions of the four model CARGs, G6PD displayed high expression in the high-risk group (Fig. 2C, Fig. S4A), whereas RPS6KB1, CYB5R4, and PIK3CA exhibited low levels of expression in the high-risk groups (Fig. 2C, Figs. S4B–S4D).

**Figure 3:**
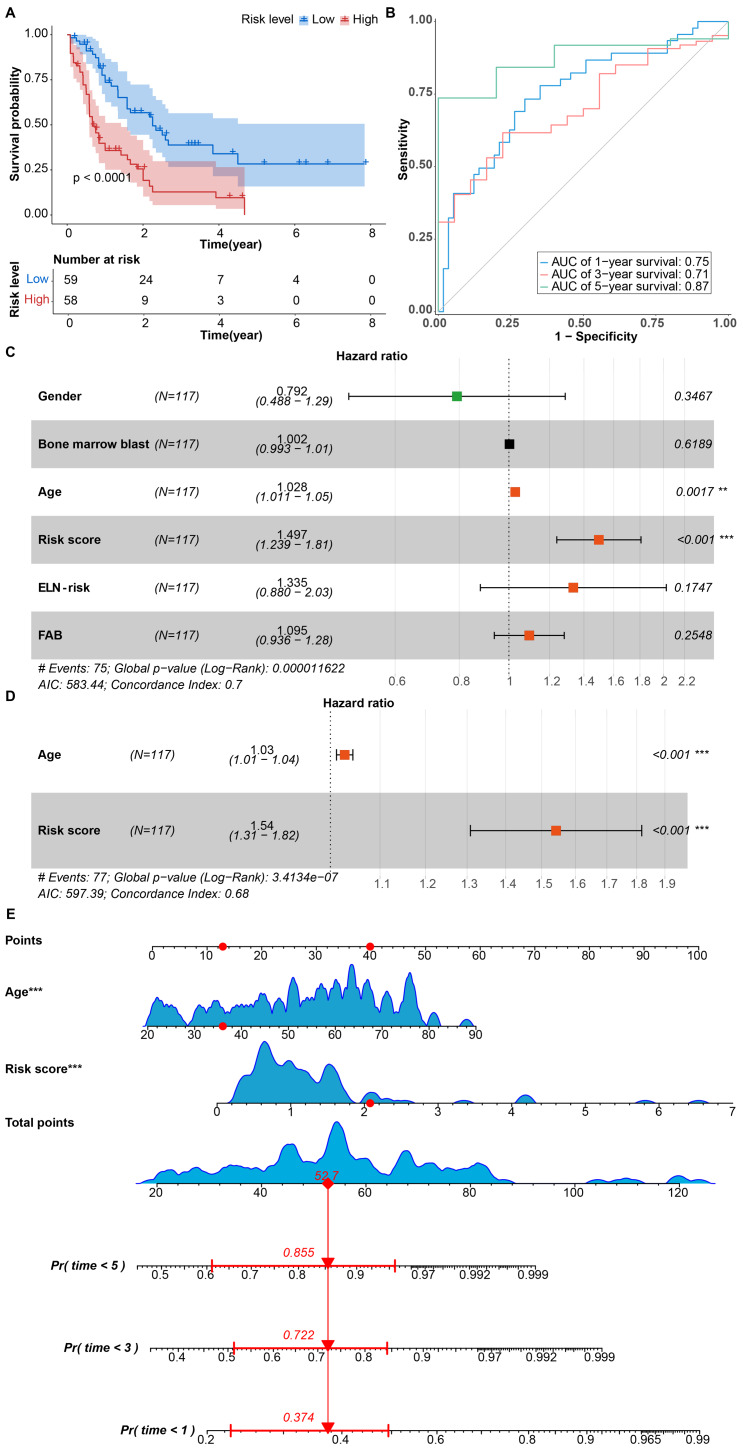
(A) Kaplan–Meier curves depicting the OS in patients grouped by risk. (B) AUC curves illustrating the performance of the CARG signature for 1, 3, and 5 years. (C) Univariate Cox regression analysis of risk scores and clinical parameters. (D) Multivariate Cox regression analysis of risk scores and age. (E) Construction of the CARG signature clinicopathologic nomogram for predicting OS for AML patients by integrating risk score and age.

Subsequently, we conducted a Kaplan–Meier analysis to validate the accuracy of the CARG signature for predicting prognosis in AML. The outcome revealed that patients in the high-risk group experienced considerably shorter OS compared to those in the low-risk group (Fig. 3A), indicating the CARG signature’s proficiency in effectively forecasting the prognosis of AML. Additionally, the area under the curve (AUC) of the CARG signature for 1-, 3-, and 5-year OS was 0.75, 0.71 and 0.87, respectively (Fig. 3B). To provides a more rigorous assessment of model generalizability, we performed bootstrap internal validation (1000 resamples) on the training cohort using the boot and rms package in R. The area under the curve (AUC) of the CARG signature for 1-, 3-, and 5-year OS was 0.75 (0.66–0.85), 0.71 (0.59–0.82) and 0.87 (0.78–0.96), respectively (Fig. S5). We conducted a univariate Cox regression analysis to evaluate the predictive independence of the CARG signature for AML patients. The analysis demonstrated a significant correlation between age and risk score with the OS of the patients (Fig. 3C). The multivariate Cox regression analysis, after adjusting for these clinical parameters, confirmed the risk score as an independent predictor for AML patients (Fig. 3D). Using 1,000 bootstrap resamples to correct for overfitting, we calculated an optimism-adjusted concordance index (C-index) of 0.676 (95% CI [0.614–0.742]) for the final multivariable Cox model. The apparent (uncorrected) C-index of 0.677 revealed minimal optimism bias (△ = 0.001), demonstrating the robustness of our internal validation approach (Table S14). To enhance the accuracy of evaluating the CARG signature, we constructed a nomogram that combines the risk score and age (Fig. 3E). The calibration curves suggested that the utility of 1- and 3-year OS could be more accurately predicted in AML patients compared with the utility of 5-year OS (Figs. S6A–S6C), indicating that the integration of our risk score and age may improve OS prediction.

### External verification of the CARG signature

**Figure 4:**
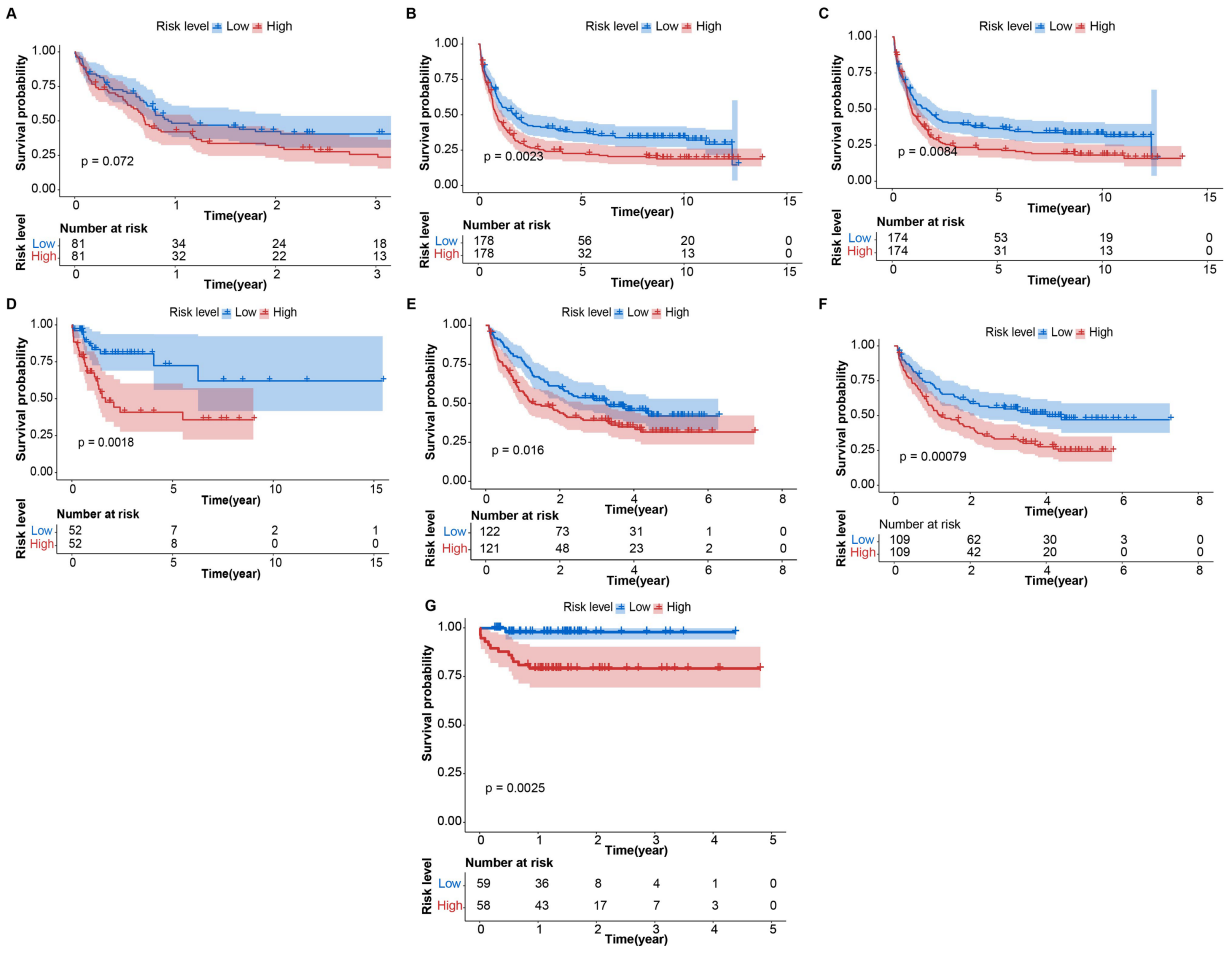
Kaplan–Meier curves of the CARG signature in external test sets (A–G) Kaplan–Meier curves of the CARG signature in the GSE12417 (GPL96), GSE37642 (GPL570), GSE37642 (GPL96), GSE71014 (GPL10558), GSE106291 (GPL18460), GSE146173 (GPL18460) datasets and our cohort.

To assess OS predictive accuracy, risk scores were computed for 1548 patients (GSE12417/GPL96, *n* = 104; GSE37642/GPL570, *n* = 243; GSE37642/GPL96, *n* = 218; GSE71014/GPL10558, *n* = 162; GSE106291/GPL18460, *n* = 356; GSE146173/GPL18460, *n* = 348; our cohort, *n* = 117) using the CARG signature. The patients were subsequently categorized into high- and low-risk groups based on the median risk score. Consistent results were observed in the seven external test sets: there was a higher proportion of patients alive in the low-risk group compared to the high-risk group (Fig. S7–S13), and patients in the high-risk group exhibited significantly shorter OS compared to those in the low-risk group (Figs. 4A–4G). The AUC values obtained from the seven external test sets provided further validation of the accuracy of the CARG signature in predicting AML prognosis (Fig. S14). Furthermore, the CARG signature was identified as an independent predictor of AML patient outcomes through both univariate and multivariate Cox regression analysis (Fig. S15–S20). Additionally, the expression levels of the 4 key genes were confirmed in the 7 test sets, revealing consistent expression patterns across these datasets (Fig. S21–S27). Overall, these findings suggest that the CARG signature has the potential to serve as an independent predictor of OS in AML patients.

### Identification and enrichment of DEGs

**Figure 5:**
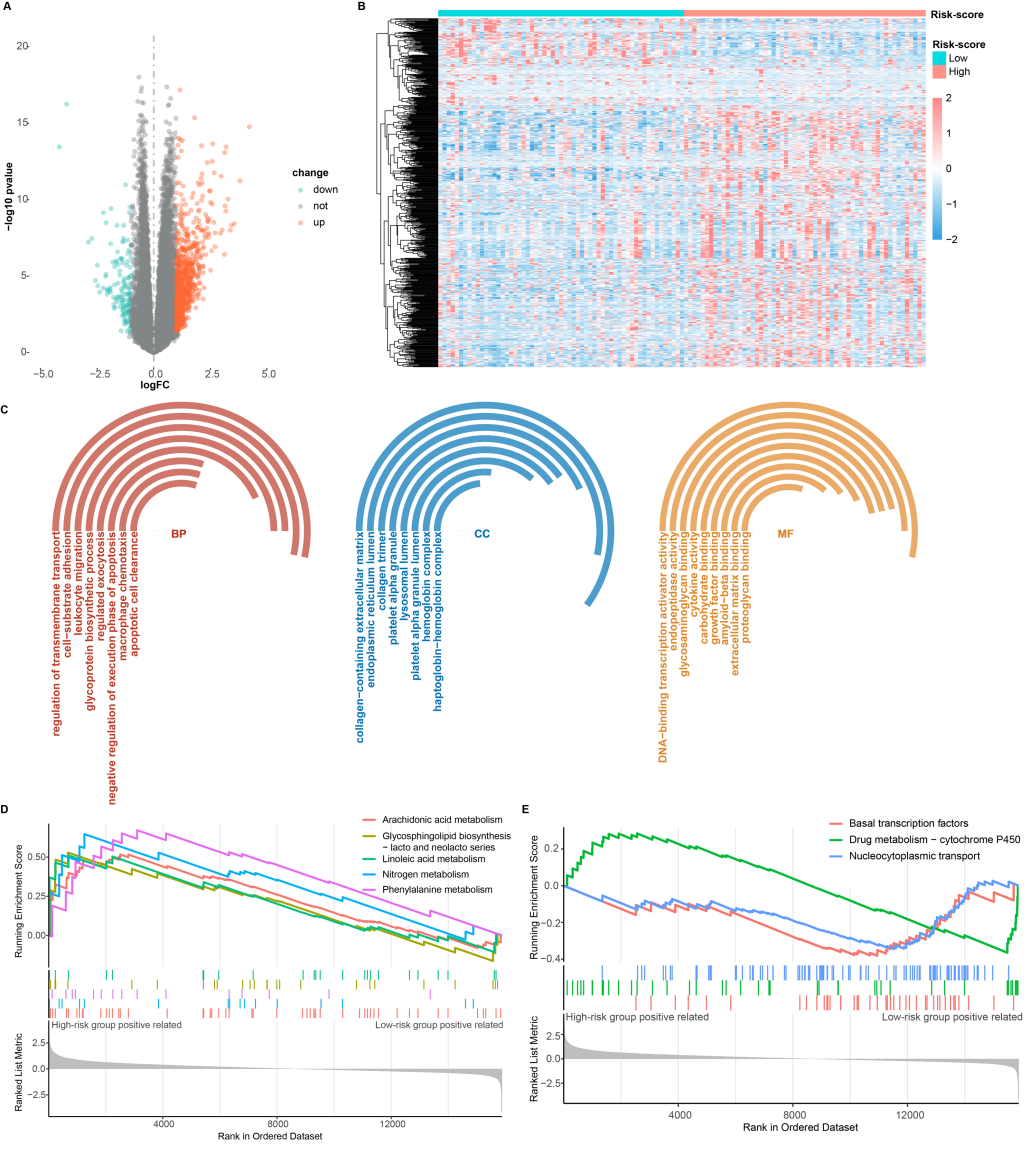
DEGs between high-risk and low-risk groups. (A) Volcano plot of the DEGs. (B) Heatmap of the DEGs. (C) GO terms significantly enriched for DEGs. (D, E) Pathways with significantly enriched DEGs.

To investigate the potential molecular mechanisms by which CARGs are involved in the regulation of OS in AML patients, we analyzed DEG patterns in the high- and low-risk groups using the DEseq2 package. We identified a total of 876 DEGs, comprising 739 up-regulated genes and 137 down-regulated genes in the high-risk group compared to the low-risk group (Figs. 5A and 5B). The GO and GSEA enrichment analyses revealed significant enrichment of these DEGs in immune responses, DNA-binding transcription activator activity, endopeptidase activity, cytokine activity, drug metabolism, and carbohydrate metabolism (Figs. 5C–5E). These results indicate that CARG plays a crucial role in immune response, DNA transcription, cytokine activity, and drug metabolism.

### Analysis of immune cell proportions and correlations in risk groups

**Figure 6:**
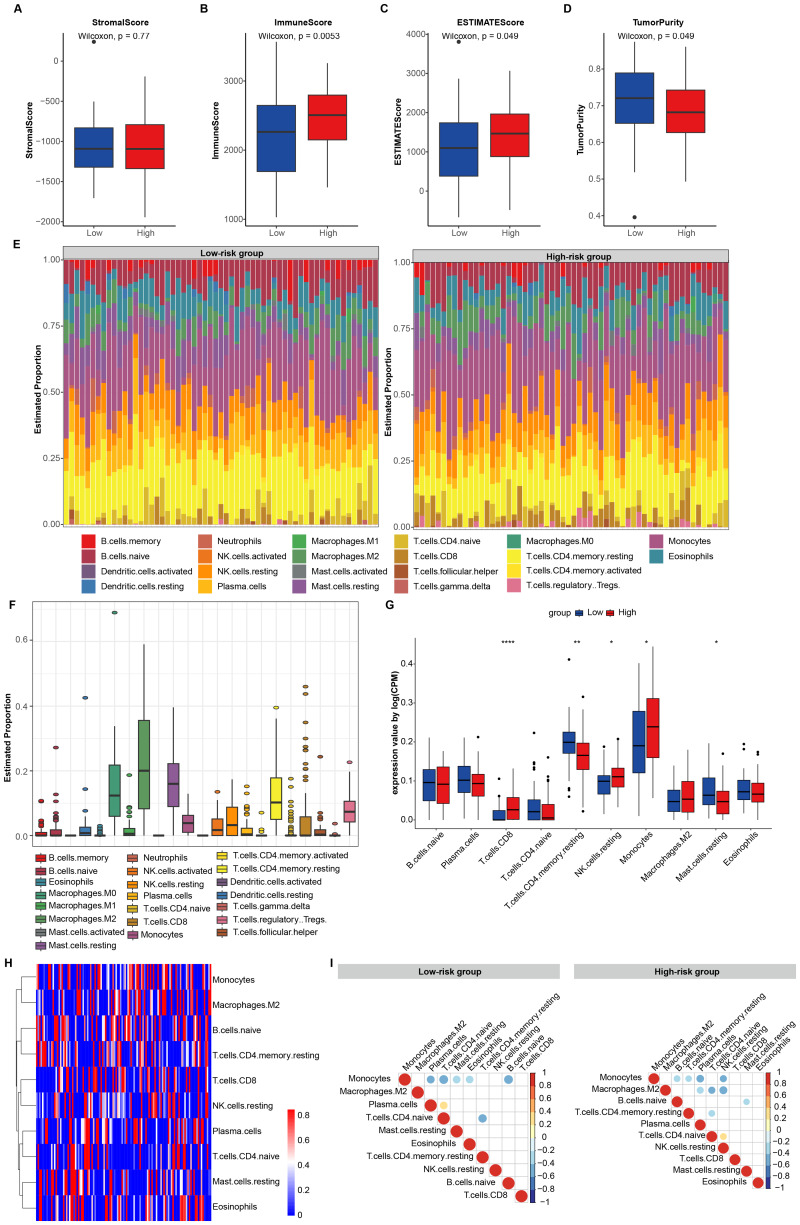
Immune cell proportion and correlations analyses in risk groups. (A) Stromal score in risk groups. (B) Immune score in risk groups. (C) Estimate score in risk groups. (D) Tumor purity in risk groups. (E) Relative proportions of immune cell in risk groups. (F) Proportions of immune cell in TCGA cohort. (G) Boxplots depicting the 22 immune cell proportions in risk groups. Significance levels are denoted as ∗ for < 0.05 and ∗∗∗ for < 0.001. (H) Heatmap of immune cell proportion in risk groups. Only the immune cells that had nonzero proportions in more than half of the samples were retained. (I) Correlations of immune cells in risk groups.

To further investigate the immune status of the risk groups, we computed the stromal score, immune score, and estimate score utilizing ESTIMATE software. Our findings revealed that immune score was notably higher in the high-risk group compared to the low-risk group, indicating a greater presence of immune infiltrating cells within the microenvironment of the high-risk group (Figs. 6A–6C). In comparison to the low-risk group, tumor purity was notably reduced in the high-risk group (Fig. 6D), indicating that the malignancy of the tumor cells in the high-risk group of AML patients is high. These findings suggest a strong correlation between the CARG signature and the immune microenvironment.

To achieve a comprehensive understanding of the AML tumor microenvironment (TME), we systematically analyzed the immune cell characteristics of 22 distinct immune cell types across all AML patients and different risk groups (Figs. 6E–6F). This analysis was conducted using the CIBERSORT algorithm with 1,000 permutations (Wang et al., 2020a; Wang et al., 2020b). We observed that the high-risk group had a notably higher proportion of CD8+ T cells, resting natural killer (NK) cells and monocytes, while simultaneously exhibiting a significantly lower proportion of resting CD4+ memory T cells and resting mast cells compared to the low-risk group (Fig. 6G). Through cluster analysis, we identified distinct groups of immune cells in AML. Specifically, monocytes and M2 macrophages, naive B cells and resting CD4+ memory T cells, CD8+ T cells and resting NK cells, plasma cells and CD4+ naive T cells, as well as resting mast cells and eosinophils were classified into separate categories (Fig. 6H).

**Figure 7:**
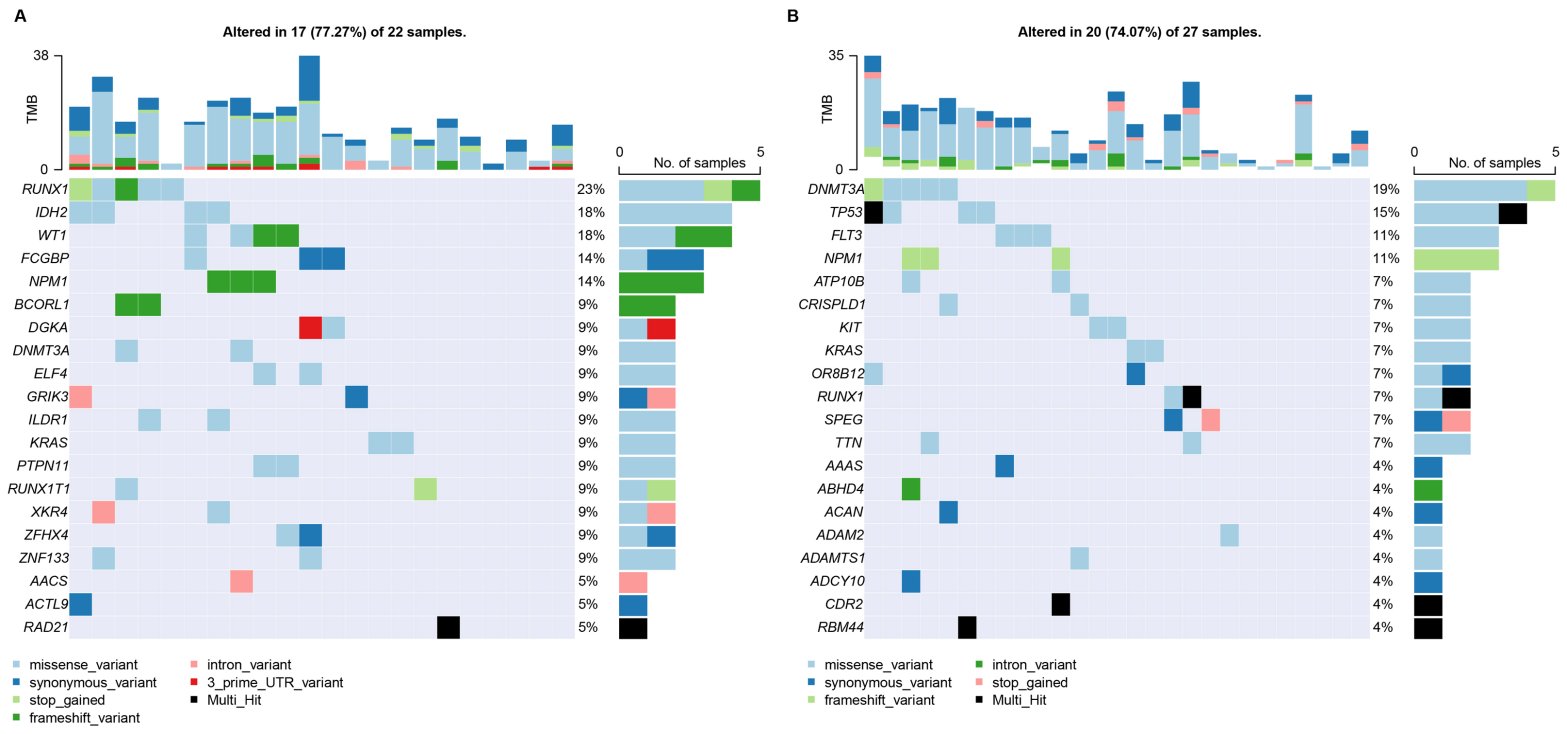
Characterization of mutations in risk groups. (A, B) Gene mutations in the low-risk and high-risk groups, respectively.

In the correlation analysis, it was observed that monocytes were negatively associated with plasma cells and naive B cells, while CD4+ memory T cells were negatively associated with CD4+ naive T cells in both groups (Fig. 6I). Additionally, in the low-risk group, monocytes were negatively associated with CD4+ naive T cells, resting mast cells, and eosinophils. In the high-risk group, monocytes were negatively associated with resting CD4+ memory T cells and resting NK cells (Fig. 6I). Plasma cells showed a positive association with CD4+ naive T cells in the low-risk group (Fig. 6I). Furthermore, M2 macrophages were negatively associated with plasma cells, CD4+ naive T cells, and resting NK cells (Fig. 7I), while naive B cells were negatively associated with resting mast cells in the high-risk group (Fig. 6I). These results confirm that the CARG signature plays significant roles in immune responses in AML.

### Characterization of mutations in risk groups

To investigate the relationship between the CARG signature and mutations in AML, we conducted an analysis of SNP profiles utilizing the Maftools R package. The gene mutations observed in AML patients classified as high-risk and low-risk exhibited distinct profiles. The prevalence of NPM1 mutation was notably elevated in both groups (Fig. 7). Mutations in RUNX1, IDH2, WT1, and FCGBP were found to be more prevalent among the top five mutations in the low-risk group (Fig. 7A), while mutations in DNMT3A, TP53, FLT3, and ATP10B were more prominent among the top five mutations in the high-risk group (Fig. 7B). These findings suggest that mutations in AML may be closely associated with the CARG signature.

### Potential drug susceptibility

**Figure 8:**
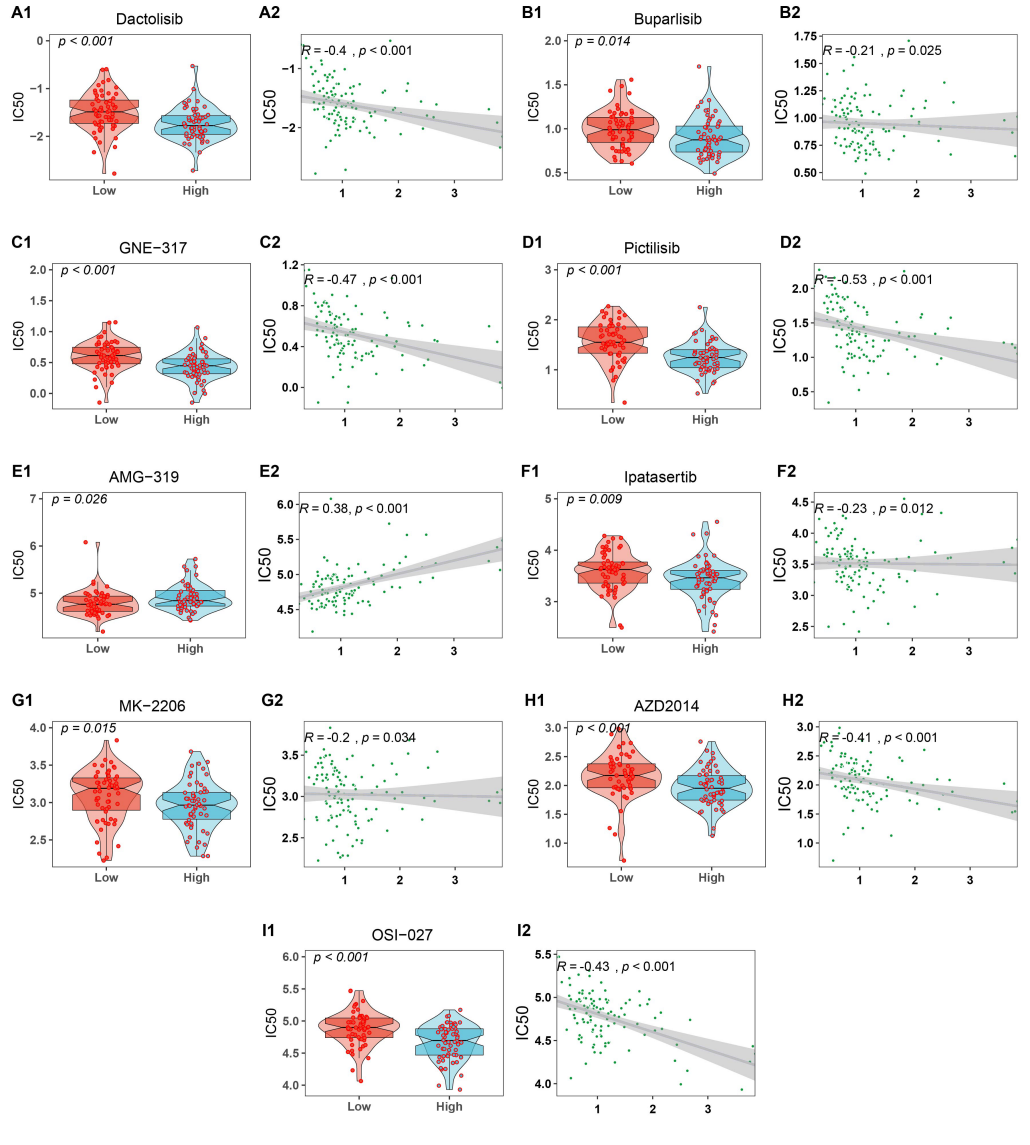
Results of potential drugs for risk groups and effect sizes. (A1–E2) Inhibitors of PI3K and effect sizes. (F1, G2) Inhibitors of AKT and effect sizes. (H1, I2) Inhibitors of mTOR and effect sizes. IC50 indicates the half maximal inhibitory concentration.

The oncoPredict R package was utilized to calculate potential clinical responses to chemotherapy based on the CARG signature. The drug response prediction function of the oncoPredict R package implements a pipeline for predicting clinical chemotherapeutic responses using solely baseline tumor gene expression data (Maeser, Gruener & Huang, 2021). Subsequently, drugs with a *P* value below 0.05 were selected for further analysis (Fig. S28). These drugs predominantly targeted the PI3K/AKT/mTOR signaling pathway (Fig. 8). Drug sensitivity associations were assessed through Spearman correlation analysis between predicted drug response and risk scores. For PI3K/AKT/mTOR pathway inhibitors, effect sizes ranged from *r* = −0.53 to −0.20 (*p* < 0.05), with the exception of AMG-319 (AKT inhibitor; *r* = 0.38, *p* < 0.001) (Fig. 8). These results suggest that higher-risk group exhibited greater sensitivity to most inhibitors in this pathway.

## Discussion

Targeted therapy and immunotherapy are vital in enhancing the prognosis for patients with AML. However, the heterogeneity in how patients respond to therapies, coupled with the frequent recurrence of the disease, leads an unsatisfactory prognosis for AML patients. Utilizing robust RNA-seq and clinical data signatures to stratify patients for accurate prognosis and drug response predictions can enhance physicians’ decision-making capabilities in selecting personalized treatment strategies. In our study, we developed a CARG signature consisting of four CARGs that accurately evaluate the overall survival of AML patients. Based on the median risk score, we stratified the patients into high and low-risk groups. The high-risk group exhibited significantly shorter OS compared to the low-risk group. We found that CARG signature served as a reliable and independent prognostic predictor of in AML through univariate Cox analysis, and this finding was validated in a total of 1,545 externally validated samples.

Through GO and GSEA enrichment analysis, we discovered that the DEGs were significantly enriched in immune responses, essentially in macrophage. M0 macrophages differentiate into M1 and M2 macrophages following polarization, with M1 macrophages exhibiting anti-tumor properties, while M2 macrophages facilitate tumor proliferation and may contribute to the immune escape of tumor cells (Bosco, 2019). High infiltration of M2 macrophages was found to be associated with poor prognosis in AML (Xu et al., 2020), and inhibiting the polarization of M2 macrophages in AML suppressed the progression of the disease (Liu et al., 2022). Further research demonstrated that inhibition of efferocytosis reduced the expression of M2 markers in leukemia-associated macrophages, such as PD-L1, PD-L2, Tim-3, CD163, and arginase-1, thereby inducing repolarization to an M1 phenotype, marked by increased levels of CD86 and HLA-DR. Ultimately, this intervention resulted in prolonged survival of mice with AML (Cruz Cruz et al., 2023). We consistently observed a higher frequency of M2 macrophages in the high-risk group (Fig. 7G). Although the difference in numbers was not statistically significant, it suggests that M2 macrophages may play a potent role in this context.

We observed a greater presence of immune infiltrating cells in the microenvironment of the high-risk group compared to the low-risk group (Fig. 7B), further confirming the close association of CARG signature with immune response. We found that the tumor purity in the high-risk group was lower than that in the low-risk group (Fig. 7D), suggesting that the tumor cells in the high-risk group were more malignant. In our observations, we found that the high-risk group exhibited a significantly higher proportion of CD8+ T cells, resting NK cells, and monocytes compared to the low-risk group. Simultaneously, the high-risk group showed a noticeably lower proportion of resting CD4+ memory T cells and resting mast cells (Fig. 7G).

From the point of view of individual cell types, it has been reported that the increase in CD8+ T cell numbers in the bone marrow plays a crucial role in anti-leukemia immunity by circulating between the intestine and bone marrow (Shi et al., 2024a; Shi et al., 2024b). Interestingly, we found an increased number of CD8+T cells in the high-risk group. The inhibitory receptor TIGIT, along with the ecto-nucleotidases CD39 and CD73, constitutes potential exhaustion markers for T cells. Brauneck and his colleagues detected an increased frequency of two distinct T-cell populations characterized by the co-expression of PD-1 or CD39 on TIGIT+CD73+CD8+ T cells in newly diagnosed and relapsed AML (Brauneck et al., 2021). This indicates that T cells in the high-risk group may exhibit characteristics of exhausted T cells in our study, thereby contributing to the adverse outcomes observed in this population. Additionally, exhausted T cells have been shown to be correlate with poor prognosis in AML (Kong et al., 2016).

We discovered that the high-risk group exhibited a significantly higher proportion of resting NK cells. In contrast to activated NK cells, the cytotoxic efficacy of resting NK cells against tumors was notably reduced (Masuyama et al., 2016). Research indicates that tumor-specific NK cells rapidly lose their anti-tumor functionality, adopt a distinctive tissue-resident phenotype, and cease actively promoting anti-tumor responses within 24 h of entering the TME (Dean et al., 2024). The aforementioned evidence implies that the anti-tumor capabilities of resting NK cells in AML may be substantially diminished.

In our study, an increase in monocyte levels was observed in the high-risk group. Cluster analysis revealed that monocytes exhibited similarities to M2 macrophages, suggesting that monocytes may share analogous anti-tumor functions with M2 macrophages. The level of monocyte count was reported significantly higher in patients with AML (Ren et al., 2022). In multiple myeloma, the presence of CD14+ monocytes impeded T-cell isolation when X-VIVO 15 basic medium was employed as the selection buffer (Wang et al., 2022). Increased monocyte counts were significantly linked to worse clinical outcomes. Moreover, elevated monocyte numbers at diagnosis are linked to faster disease progression, and monocytes support the survival and growth of chronic lymphocytic leukemia (CLL) cells, making them potential therapeutic targets for CLL (Friedman et al., 2016; Giannoni et al., 2022). Consistently, a high percentage of Tie2-expressing monocytes was strongly associated with unfavorable prognostic markers, such as ZAP-70, CD38, deletions of 17p and 11q, and IGHV mutational status in CLL. Additionally, patients who did not respond to first-line therapy exhibited significantly higher percentages of circulating Tie2-expressing monocytes compared to those who did respond. Moreover, a high percentage of Tie2-expressing monocytes was correlated with shorter OS and a decreased time to treatment (Woś et al., 2021). These findings suggest that monocytes play a significant role in shaping the inhibitory microenvironment of AML.

CD4+ T cells play a crucial role in tumor immunity (Speiser et al., 2023). Memory CD4+ T cells, once activated, can persist for a long time and quickly respond to re-exposure of the same antigen, which allows CD4+ T cells to have a durable immune response to the tumor and quickly launch an attack when needed (Kruse et al., 2023; Künzli & Masopust, 2023; Speiser et al., 2023). We observed a significant reduction in resting CD4+ T cells in the high-risk group, further suggesting the existence of an inhibitory immune microenvironment in this patient population.

Mast cells, once considered a neglected element of the TME have recently been identified as key contributors to tumor progression (Shi et al., 2024a; Shi et al., 2024b). Their presence is significantly correlated with poor prognosis in patients suffering from prostate cancer and a range of other solid tumors (Teng et al., 2021; Xie et al., 2021). Mast cells are pivotal factors influencing anti-tumor immunity and cancer prognosis (Lichterman & Reddy, 2021). Interestingly, a marked reduction in mast cells was observed in the high-risk group, which could adversely affect the growth and survival of tumor cells within this cohort. Our immune-related analysis revealed that the CARG signature may be crucial for AML understanding the immune landscape.

Concurrently, alterations in immune cell numbers significantly influence the remodeling of the bone marrow microenvironment. However, a singular shift in immune cell count may not adequately depict the actual condition of the bone marrow microenvironment. Therefore, a comprehensive analysis of immune cell function and cell–cell interactions is essential to accurately reflect the true state of the bone marrow microenvironment and deserves further investigation.

ELN risk classification, cytogenetic categories, and key molecular markers (*e.g.*, NPM1, FLT3-ITD/TKD, IDH1/2, TP53, DNMT3A) play key role in AML prognosis (Shimony, Stahl & Stone, 2025). The inclusion of these factors in our study would substantially strengthen the prognostic analysis in AML. However, due to the different focuses of each study and the high heterogeneity of the data, we face significant limitations when applying this method uniformly to all datasets. First, the TCGA training set contains ELN classification and key molecular markers (*e.g.*, NPM1, FLT3-ITD/TKD, IDH1/2, TP53, DNMT3A), but lacks cytogenetic data. Notably, among the 117 non-APL samples, only 49 possessed complete mutation profiles, and just 34 (29.1%, Table S15) had both mutation data and RNA-seq information. This limited sample size raises concerns about the reliability of modeling efforts, as small cohorts may lead to overfitting or reduced statistical power. Additionally, in validation datasets, GSE12417 (GPL96) and GSE71014 (GPL10558) are restricted to normal karyotypes, lacking other cytogenetic categories, mutation profiles, or ELN risk classification. GSE37642 (GPL570, GPL96) and GSE106291 (GPL18460) only provide RUNX1-mutation and RUNX1-RUNX1T1-fusion data, with no additional cytogenetic or molecular marker information. Among external datasets, only GSE146173 contained any of these clinical variables. Missing data patterns varied substantially across cohorts (Table S16), which has led to our inability to conduct large-scale verification of the model’s reliability. Therefore, cytogenetic classification and key molecular markers (*e.g.*, NPM1, FLT3-ITD/TKD, IDH1/2, TP53, DNMT3A) were not incorporated into the Cox models in this study. We anticipate future high-quality studies with comprehensive data and larger sample sizes, which will facilitate further refinement of these prognostic models.

Genetic mutations are highly prevalent in AML. Although the paucity of mutation-annotated cases in the training set precluded comprehensive modeling, we conducted a systematic comparison of mutational profiles between high- and low-risk group. Many studies have demonstrated that DNMT3A mutations are correlated with an unfavorable prognosis in patients (Wang et al., 2020a; Wang et al., 2020b; Narayanan et al., 2021; Huang, Cai & Li, 2024). Consistently, mutations in DNMT3A were discovered more prevalent in the high-risk group in our study (Fig. 8B). In both *in vitro* and *in vivo* experiments, DNMT3A-mutated AML cells were found to diminish the polarization of M1 macrophages and to resist their cytotoxic effects. In xenograft models, the tumor volume in the DNMT3A-mutated group was significantly larger, and the proportion of M2 macrophages was notably higher (Que et al., 2021). Among high-risk patients, mutations in TP53 were the second most commonly observed type of mutation (Fig. 8B). AML with TP53 mutation is indeed considered one of the most lethal types of cancer and is associated with an extremely poor prognosis (Man et al., 2023). It has been reported that AML patients with TP53 mutations exhibit significantly reduced numbers of bone marrow-infiltrating OX40+ cytotoxic T cells and helper T cells, as well as a decrease in ICOS+ and 4-1BB+ NK cells. Additionally, there is an expansion of highly immunosuppressive regulatory T cells and myeloid-derived suppressor cells in cases with TP53 mutations (Sallman et al., 2020). Therefore, the above evidence suggests that patients in high-risk groups may be more susceptible to immune evasion.

The ‘oncoPredict’ R package was utilized to evaluate the clinical drug response in patients with AML. Notably, risk groups demonstrated divergent responses to chemotherapy agents. These drugs primarily targeted the PI3K/AKT/mTOR signaling pathway. These inhibitors proved effective for AML, with most exhibiting lower IC50 values in high-risk patients. This observation suggests that targeting the PI3K signaling pathway may present a novel therapeutic approach for the treatment of AML.

## Conclusions

We developed a CARG prognostic signature consisting of four genes in patients with AML. The OS in the high-risk group was significantly shorter. The CARG prognostic signature is strongly linked to immune responses, and immunosuppression could be prevalent in high-risk patients. The findings regarding potential drugs for the risk groups showed that inhibitors of the PI3K/AKT/mTOR signaling pathway were effective. The new risk model based on CARGs proposed in our study holds promise for prognostic classifications in AML, potentially offering fresh insights for the development of precise targeted cancer therapies.

## Supplemental Information

10.7717/peerj.21168/supp-1 Supplemental Information 1 Supplementary Figures

10.7717/peerj.21168/supp-2 Supplemental Information 2 Supplementary Tables

10.7717/peerj.21168/supp-3 Supplemental Information 3 Supplementary Table codebook

10.7717/peerj.21168/supp-4 Supplemental Information 4 R codes

10.7717/peerj.21168/supp-5 Supplemental Information 5 MIQE checklist

10.7717/peerj.21168/supp-6 Supplemental Information 6 Raw Data

## Abbreviations

AML: acute myeloid leukemia
CARGs: carbohydrate metabolism and autophagy-related genes
OS: overall survival
DEGs: differentially expressed genes
OXPHOS: oxidative phosphorylation
HBP: hexosamine biosynthetic pathway
PPP: pentose phosphate pathway
TCGA: the cancer genome atlas
LASSO: least absolute shrinkage and selection operator
GEO: gene expression omnibus
SNP: single nucleotide polymorphism
KEGG: Kyoto Encyclopedia of Genes and Genomes
HADb: human autophagy database
ROC: operating characteristic
GO: Gene Ontology
GSEA: gene set enrichment analysis
PBMCs: peripheral blood mononuclear cells
AUC: area under the curve
NK: natural killer
TME: tumor microenvironment
CLL: chronic lymphocytic leukemia

## Additional Information and Declarations

### Data Availability

The following information was supplied regarding data availability: The raw measurements are available in the Supplemental Files.
