# Supplementary material for: Carbohydrate metabolism and autophagy signature predicts prognosis and immune microenvironment of acute myeloid leukemia
Source: PeerJ. 2026 May 22;14:e21168. doi: 10.7717/peerj.21168 (PMC13200625; doi:10.7717/peerj.21168)
Supplement: Supplemental Information 1 [file peerj-14-21168-s001.docx]

**Supplementary figures for** **carbohydrate metabolism and autophagy signature predicts prognosis and immune microenvironment of acute myeloid leukemia**

**Qingchun Shen^b,1^, Lan Xiao^a,1^, Jing Liu^a^, Miao Zhang^a^, Jiabo Ding^b^, Ling Guo^a^, Qulian Guo^a^, Jing Guo^c^, Tingting Leng ^a^, Wenjun Liu^a,*^, You Yang^a, *^**

^a^ Department of Pediatrics (Children Hematological Oncology), Birth Defects and Childhood Hematological Oncology Laboratory, The Affiliated Hospital of Southwest Medical University, Sichuan Clinical Research Center for Birth Defects, Luzhou, Sichuan, China

^b^ Institute of Animal Science of CAAS，No. 2, Yuanmingyuan West Road, Haidian District, Beijing, China

^c^ Department of Hematology, The Affiliated Hospital of Southwest Medical University. Luzhou, Sichuan, China

^1^ These authors have contributed equally to this work.

* Correspondence

Wenjun Liu: [wenjun_liu@swmu.edu.cn](mailto:wenjun_liu@swmu.edu.cn);

You Yang: [youyang091@swmu.edu.cn](mailto:youyang091@swmu.edu.cn).


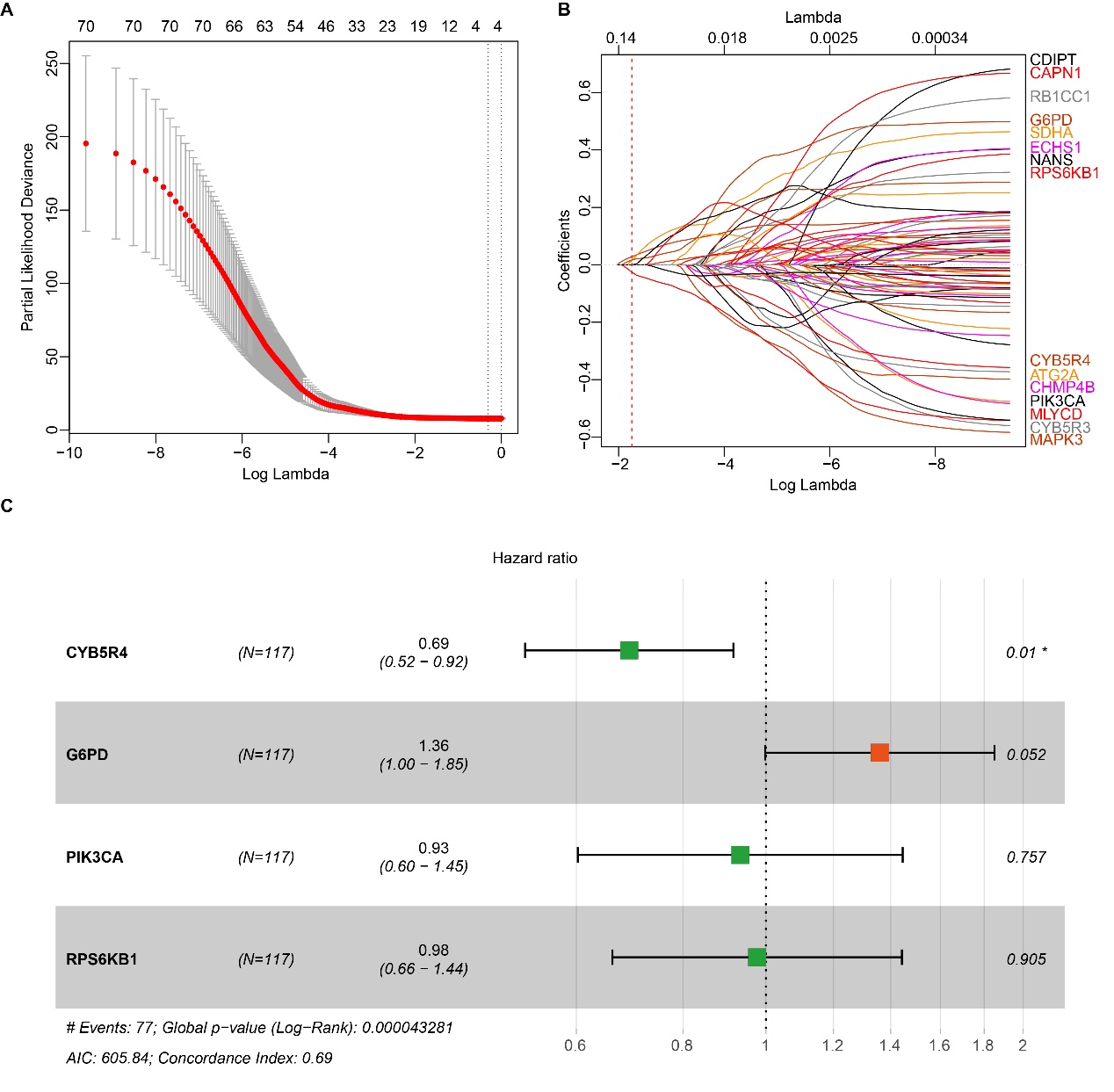


**Supplementary Figure S1. Identification and development of a CARG signature for OS in AML training set**

1. Cross-validation for tuning parameter selection in the proportional hazards model. (B) LASSO coefficient spectrum of 4 CARGs in AML. (C) The hazard ratio of 4 model CARGs. HR indicates hazard ratio; CI indicates confidence interval.


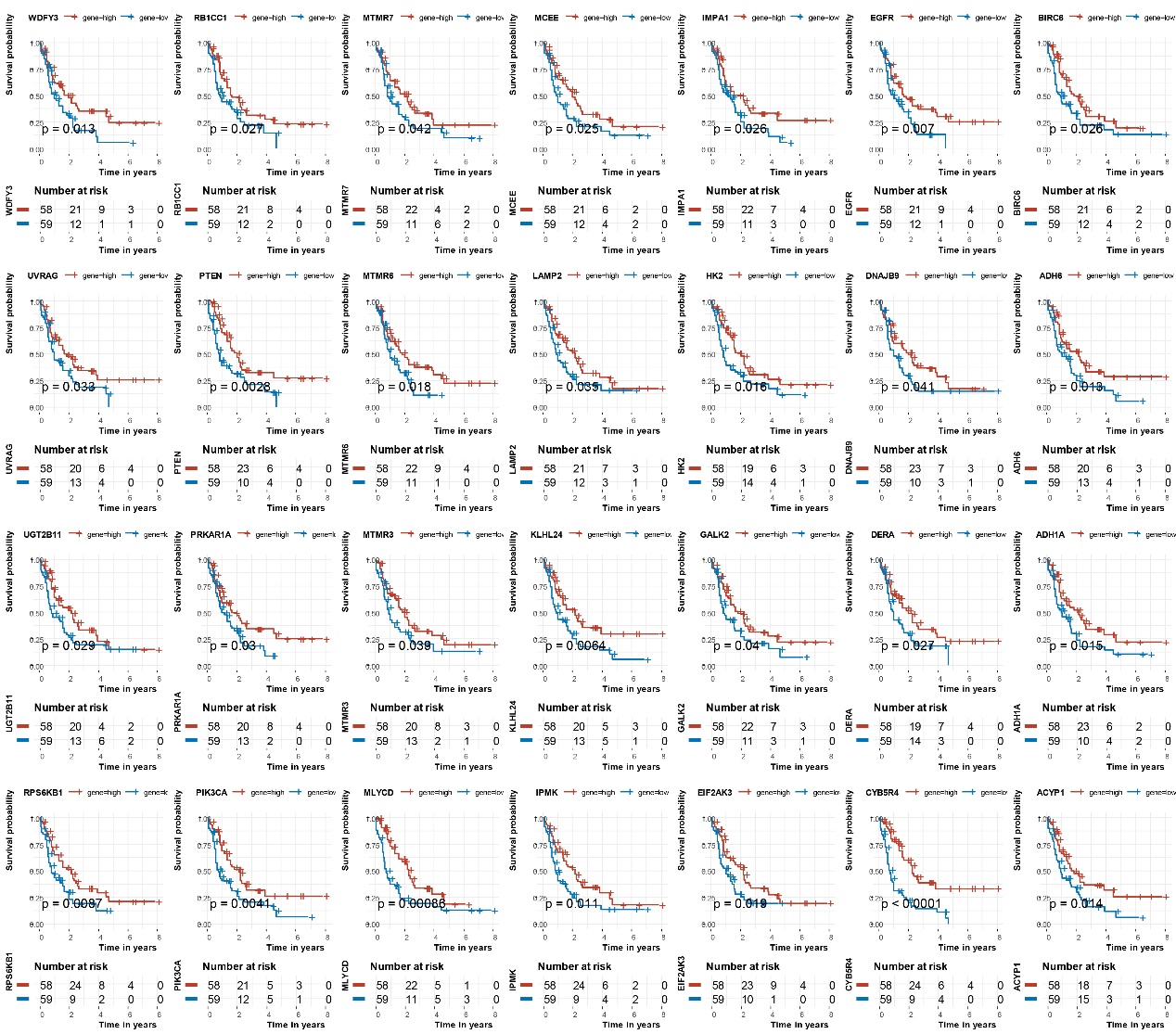


**Supplementary Figure S2.** Kaplan-Meier curves of OS for 28 protective factors

Kaplan-Meier analyses of overall survival were performed based on clinical and molecular data from 117 AML patients. The patients were stratified based on mRNA levels in the primary tumor, with those exceeding the median level represented in red, and those below the median level in blue. P values obtained from the log-rank test are provided.


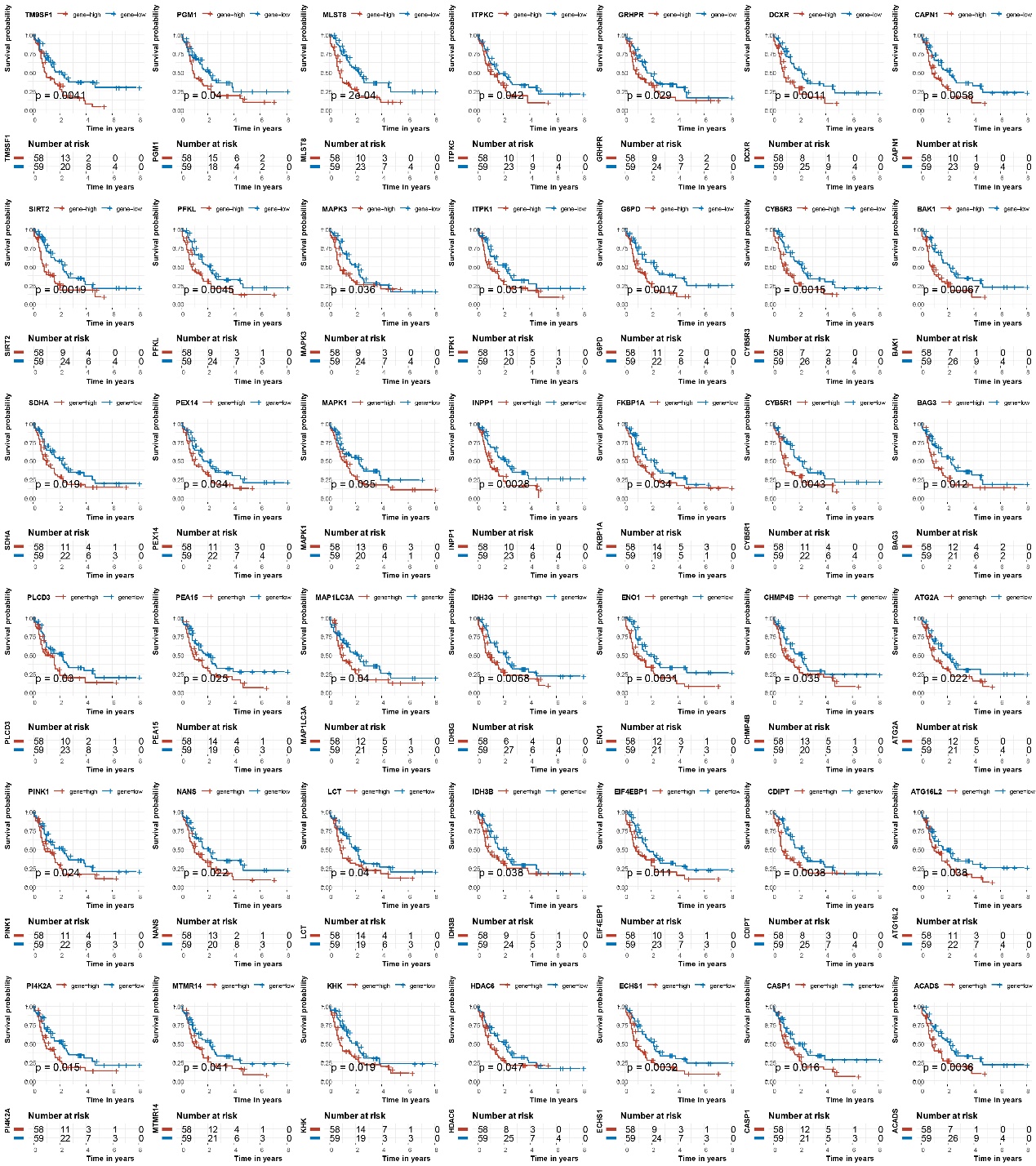


**Supplementary Figure S3.** Kaplan-Meier curves of OS for 42 risk factors

Kaplan-Meier analyses of overall survival were performed based on clinical and molecular data from 117 AML patients. The patients were stratified based on mRNA levels in the primary tumor, with those exceeding the median level represented in red, and those below the median level in blue. P values obtained from the log-rank test are provided.


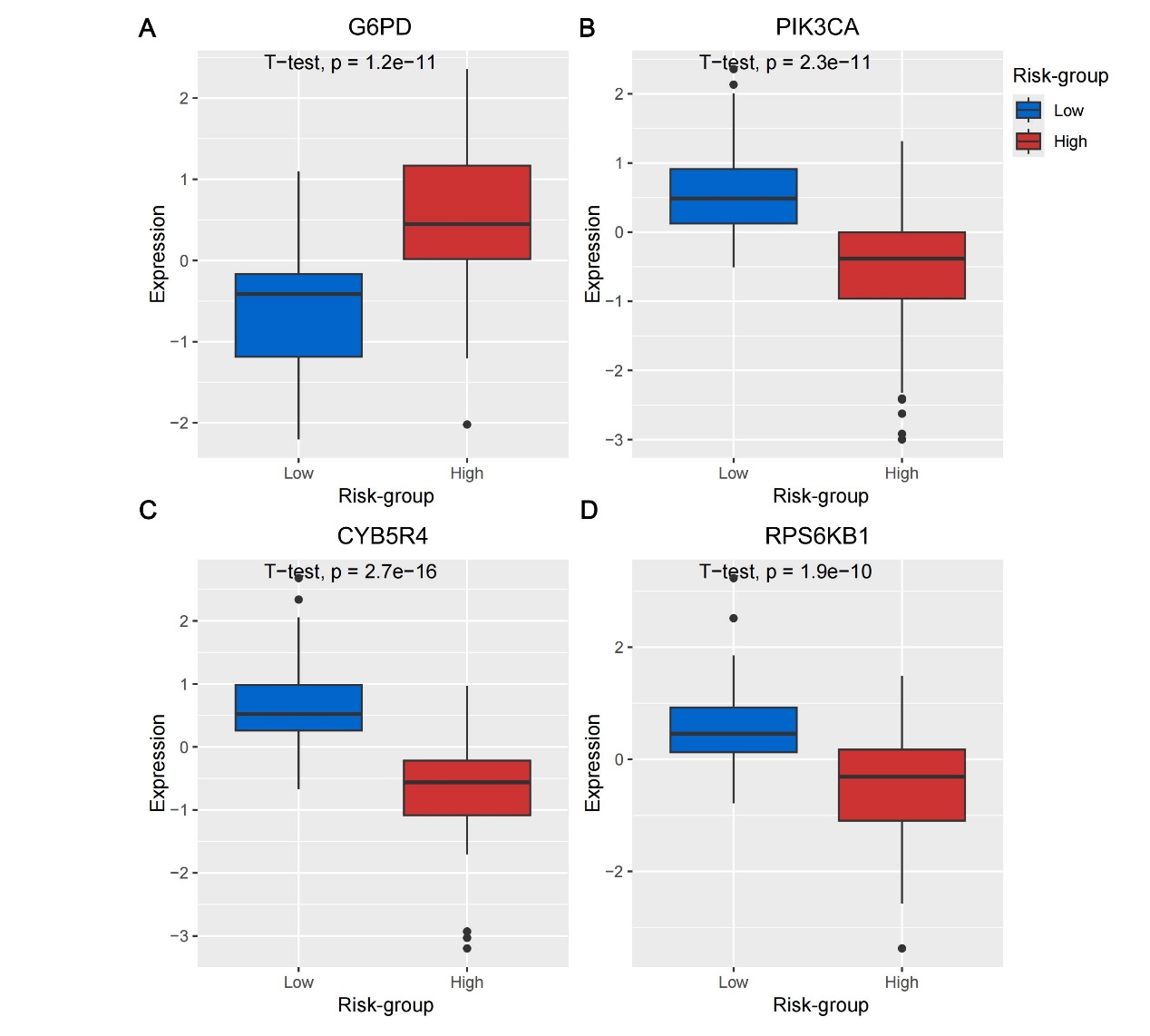


**Supplementary Figure S4.** Relative mRNA expression levels of 4 CARGs in high and low risk groups (training set)


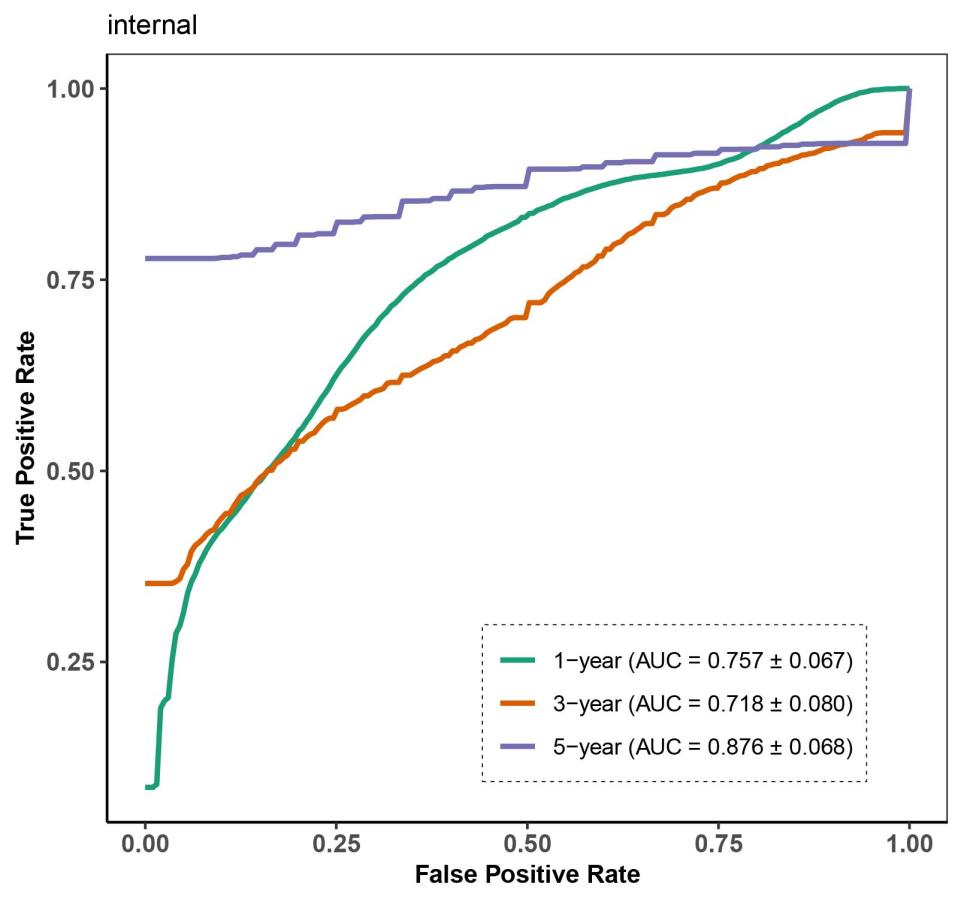


**Supplementary Figure S5.** Time-dependent AUCs at 1-, 3-, 5-year survival (bootstrap, 1000 resamples).


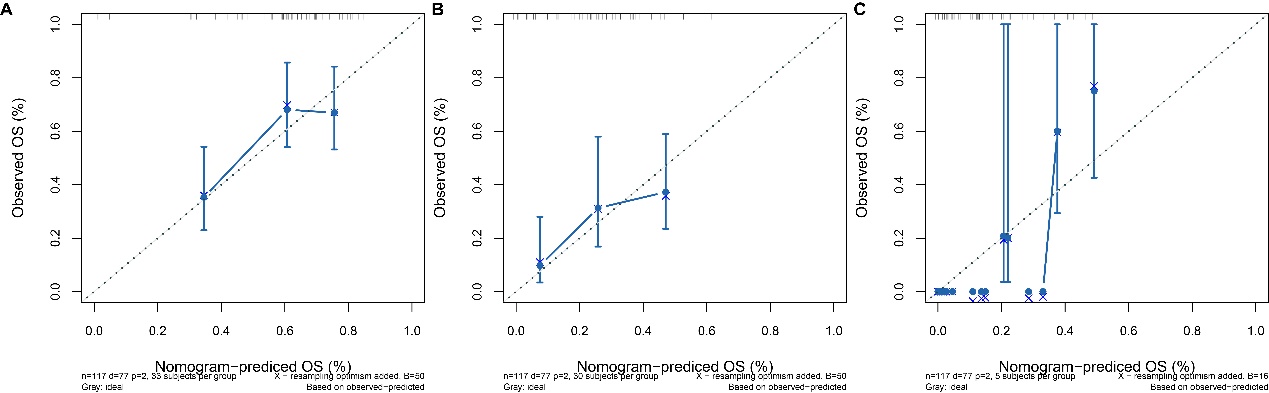


**Supplementary Figure S6.** Calibration curves of the CARG clinicopathologic nomogram-predicted and observed 1-, 3- and 5-year survival of AML patients.

The dashed line represents the ideal performance, while the actual performance of the CARG signature is depicted by the blue lines.


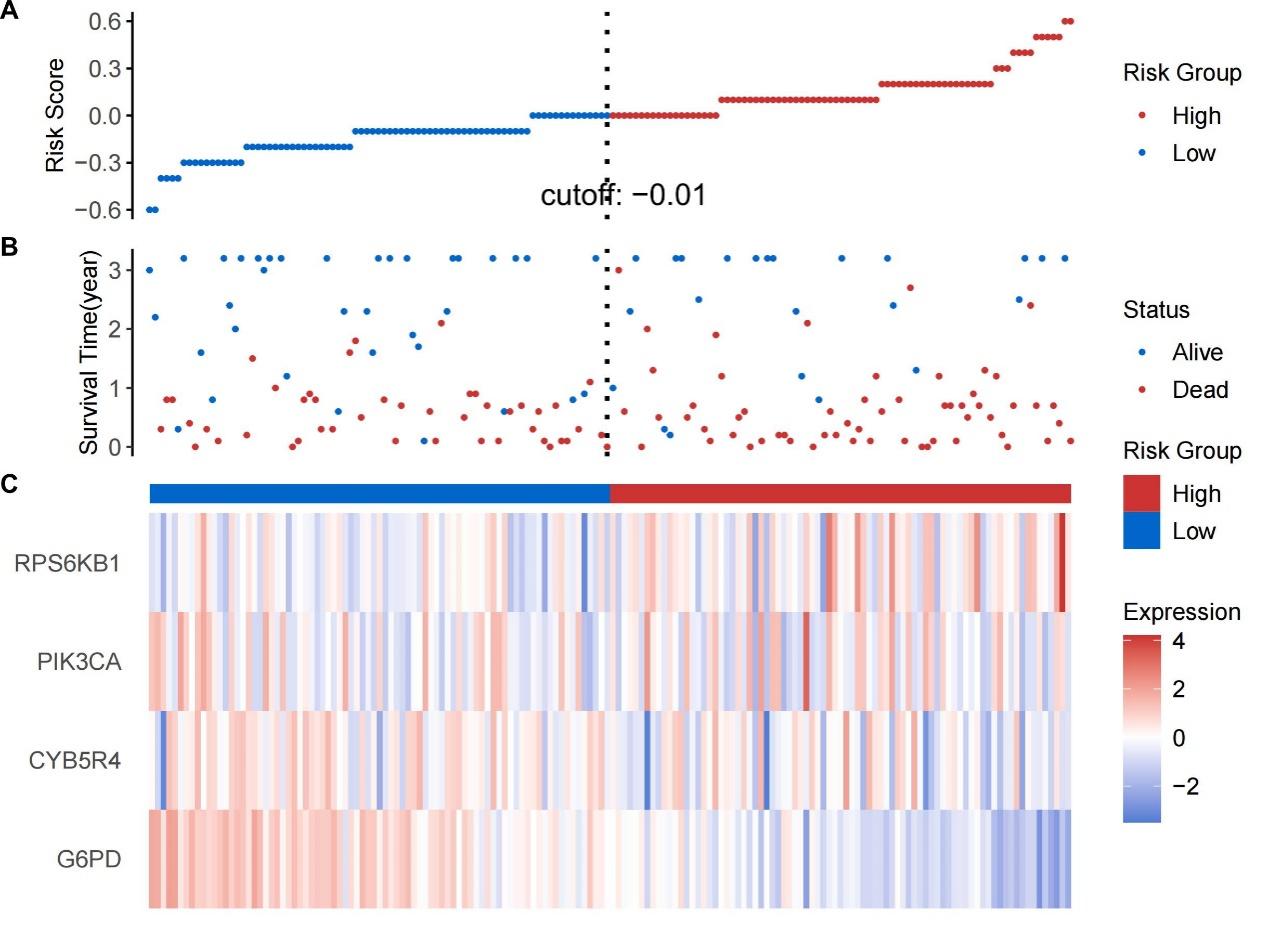


**Supplementary Figure S7.** Prognostic analysis of the CARG signature in the test set (GSE12417, GPL96, n=104)

(A) Categorization of risk groups according to patients’ risk scores. (B) Patients’ survival status in conjunction with their respective risk scores. (C) The expression levels of the 4 model CARGs in the high- and low-risk groups.


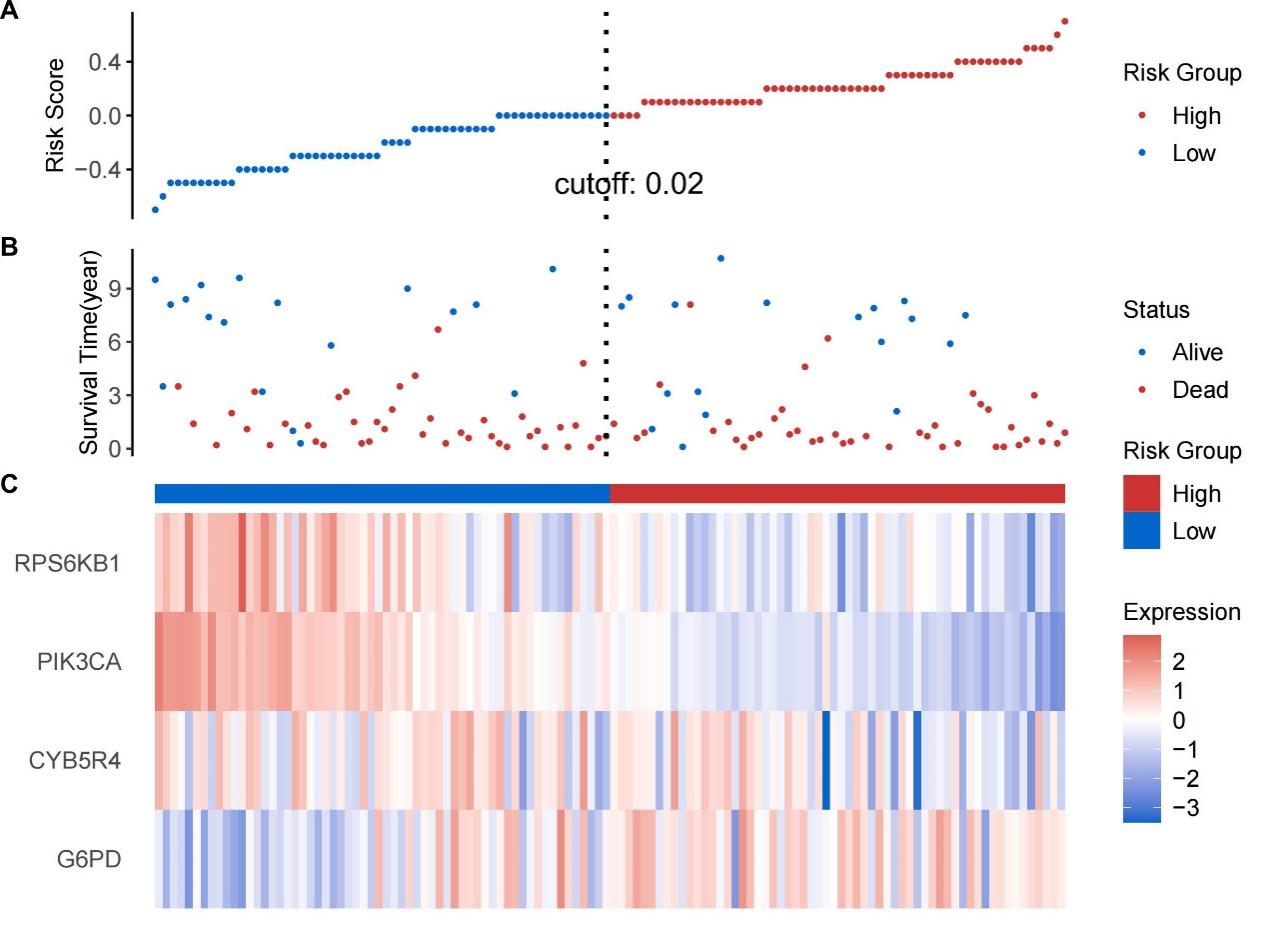


**Supplementary Figure S8.** Prognostic analysis of the CARG signature in the test set (GSE37642, GPL570)


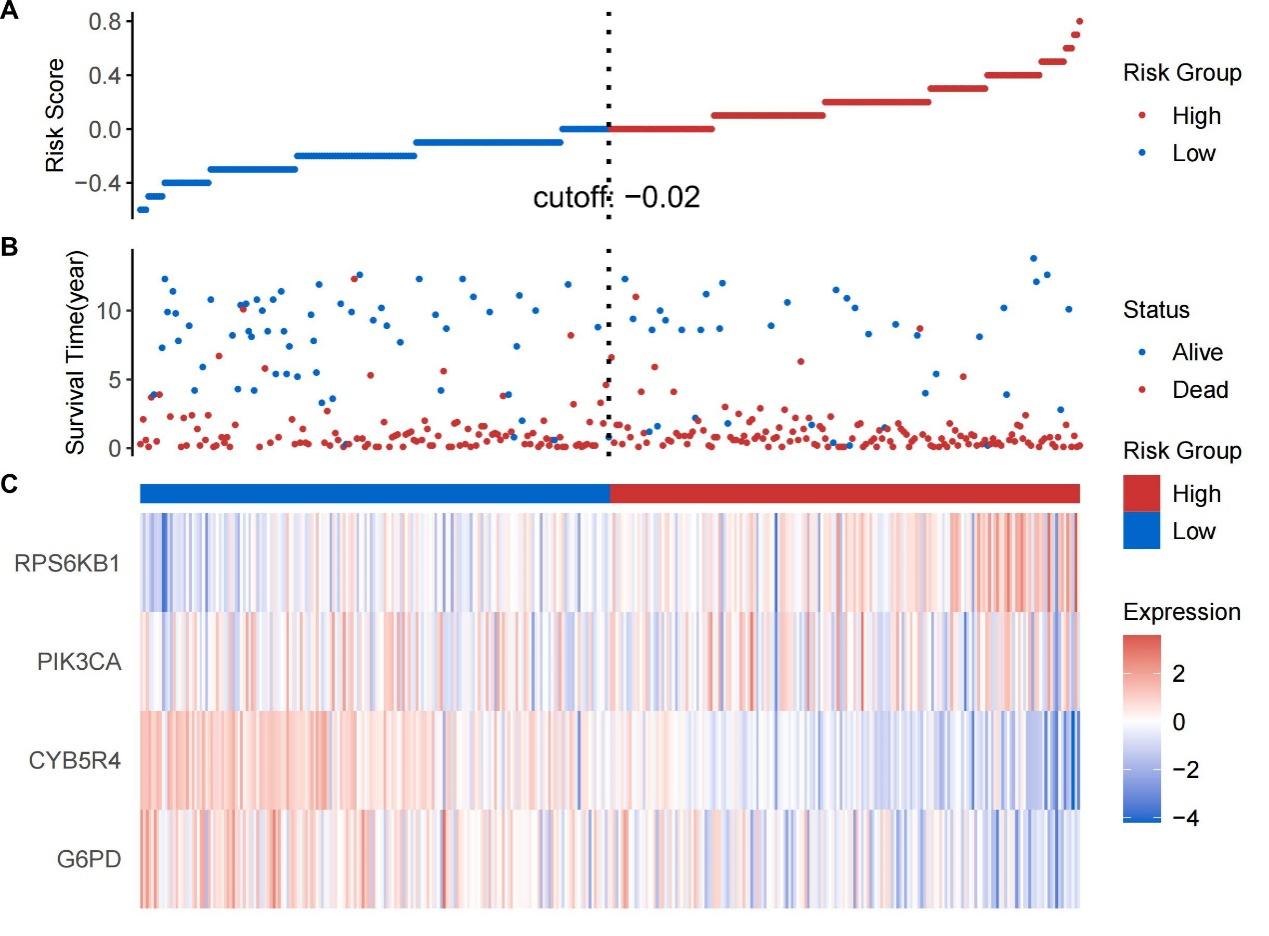


**Supplementary Figure S9.** Prognostic analysis of the CARG signature in the test set (GSE37642, GPL96)


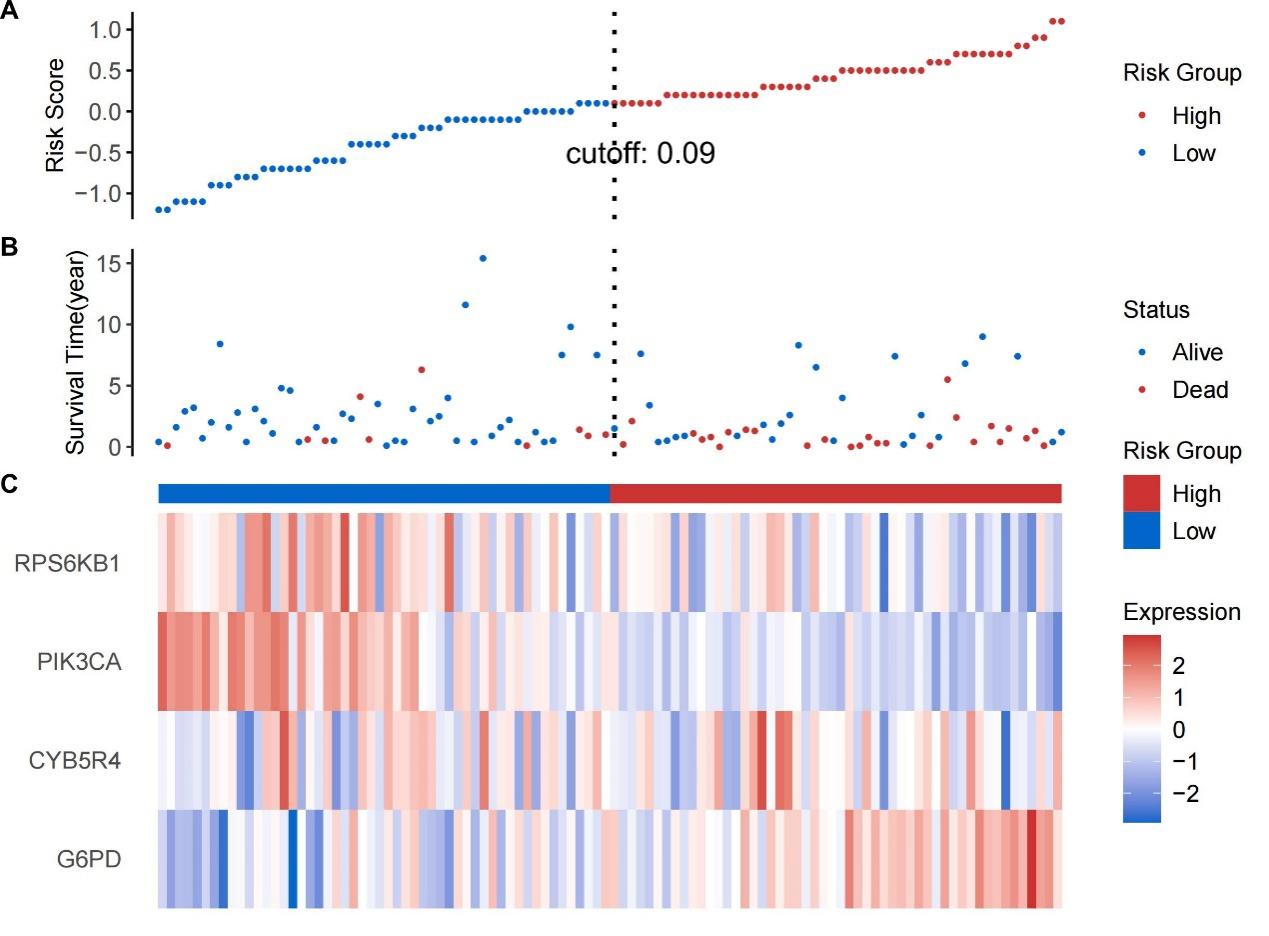


**Supplementary Figure S10.** Prognostic analysis of the CARG signature in the test set (GSE71014, GPL10558)


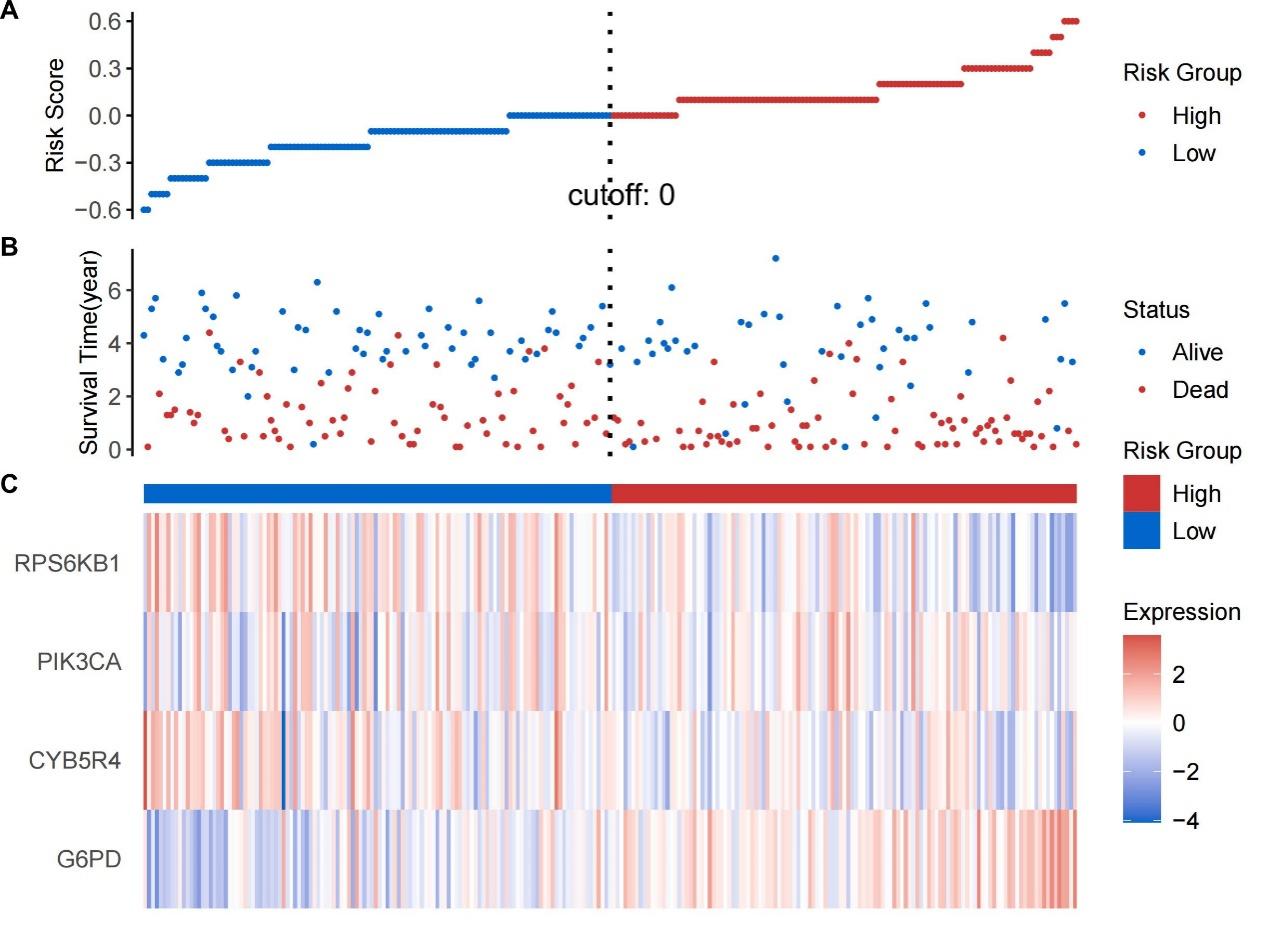


**Supplementary Figure S11.** Prognostic analysis of the CARG signature in the test set (GSE106291, GPL18460)


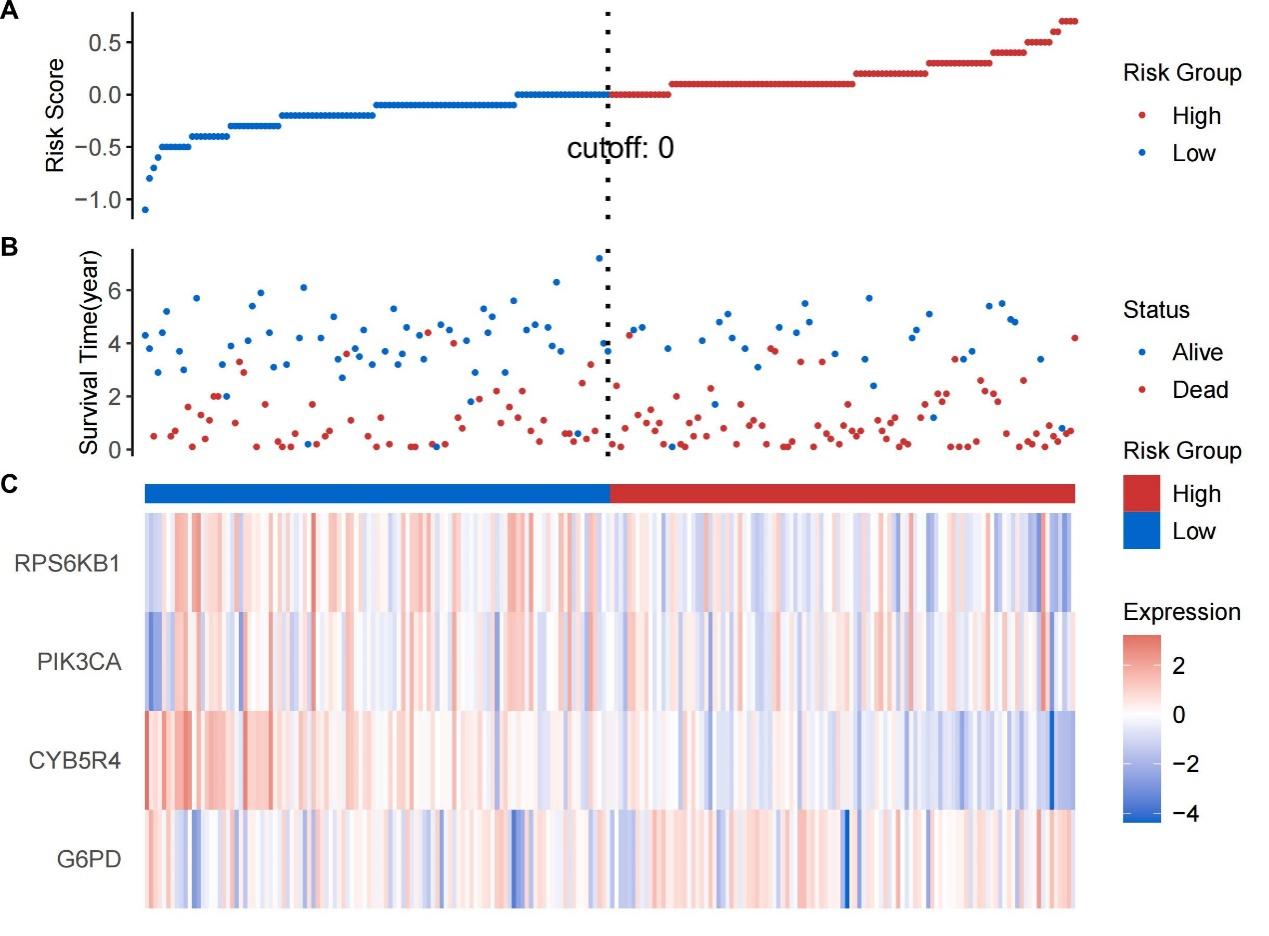


**Supplementary Figure S12.** Prognostic analysis of the CARG signature in the test set (GSE146173, GPL18460).


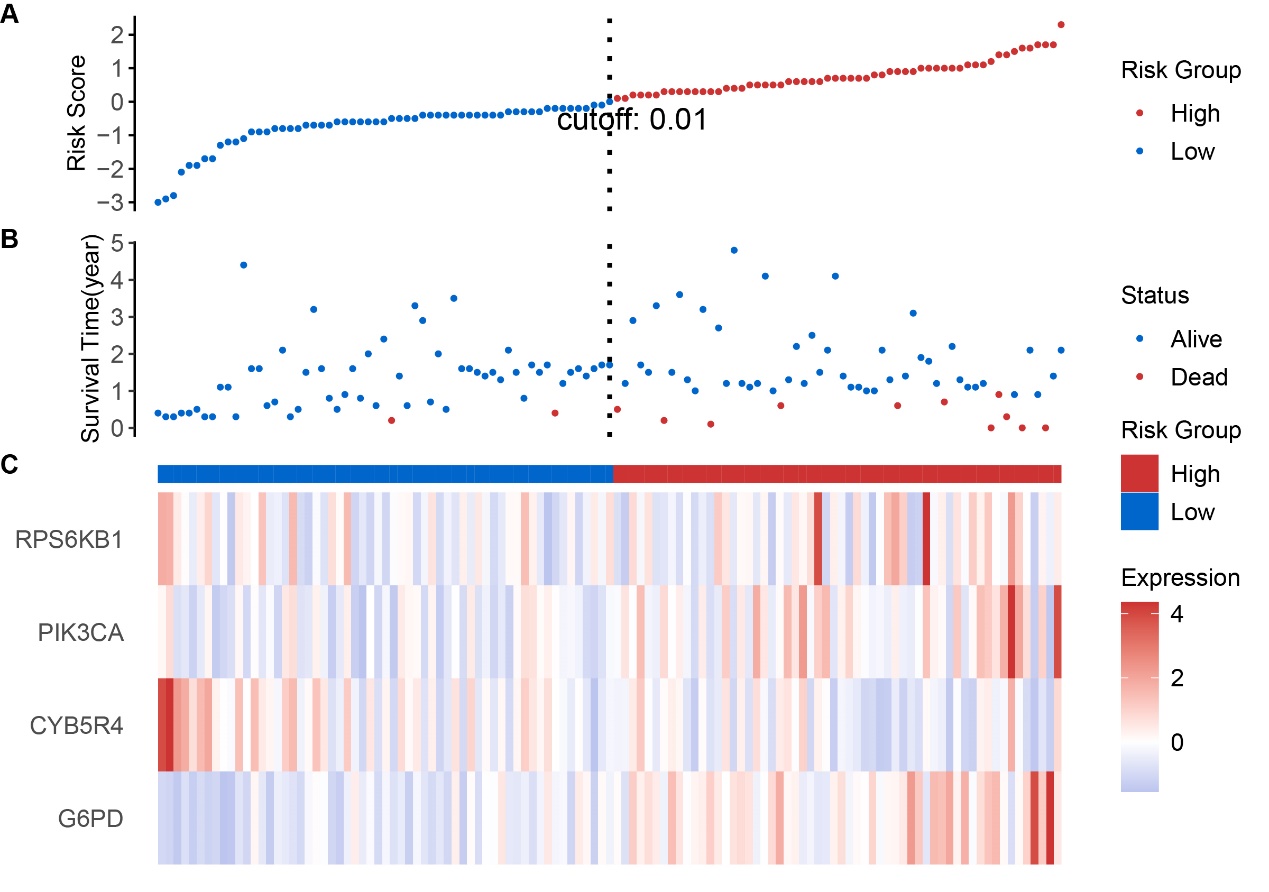


**Supplementary Figure S13.** Prognostic analysis of the CARG signature in the test set (Our cohort).


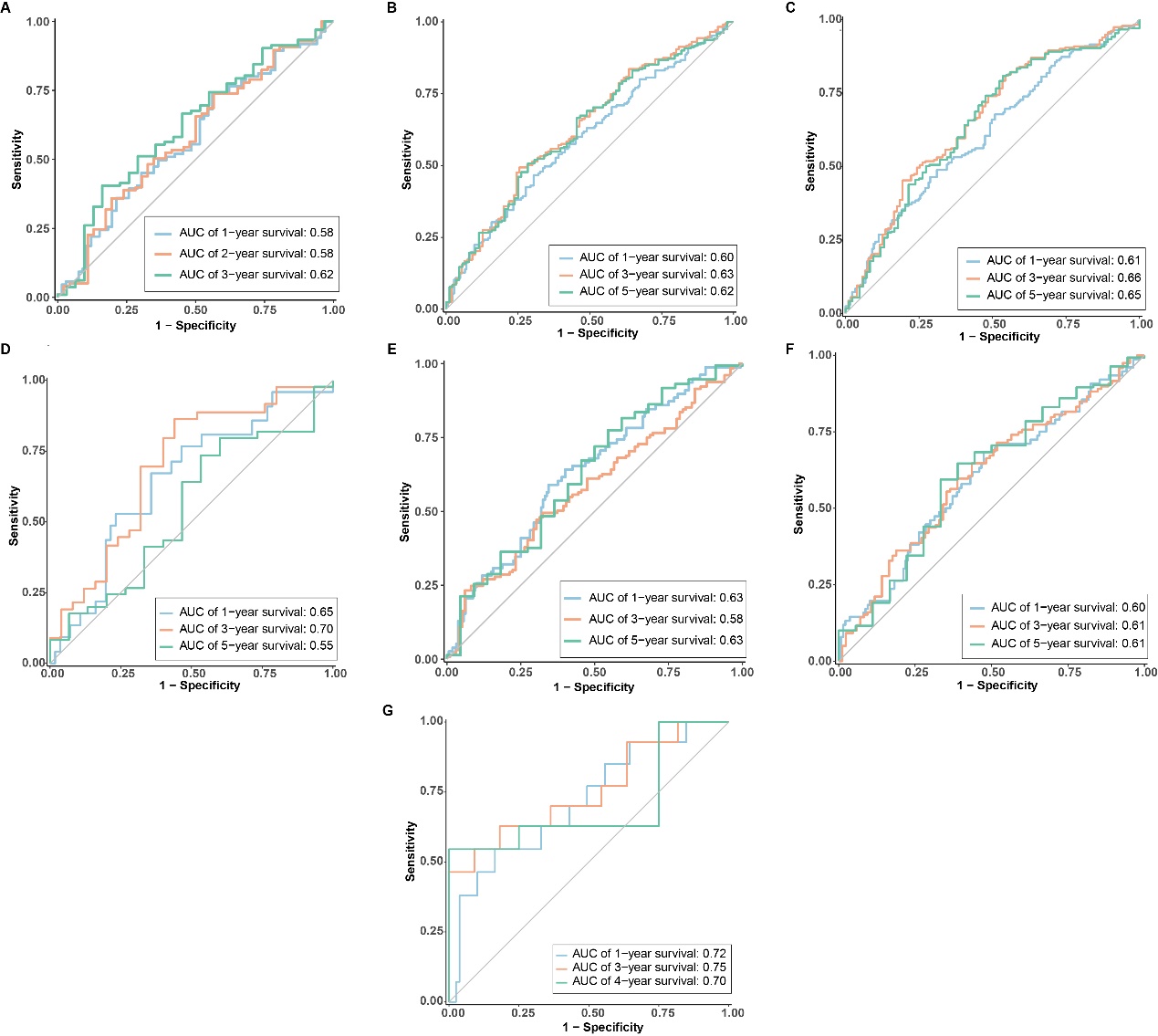


**Supplementary Figure S14. ROC curves of the CARG signature in external test sets.**

(A) The ROC curves of the CARG signature for 1-, 2-, and 3-year in GSE12417 (GPL96) dataset. (B-F) The ROC curves of the CARG signature for 1-, 3-, and 5-years in the GSE37642 (GPL570), GSE37642 (GPL96), GSE71014 (GPL10558), GSE106291 (GPL18460), GSE146173 (GPL18460) datasets. (G) The ROC curves of the CARG signature for 1-, 3-, and 4- year in our cohort.


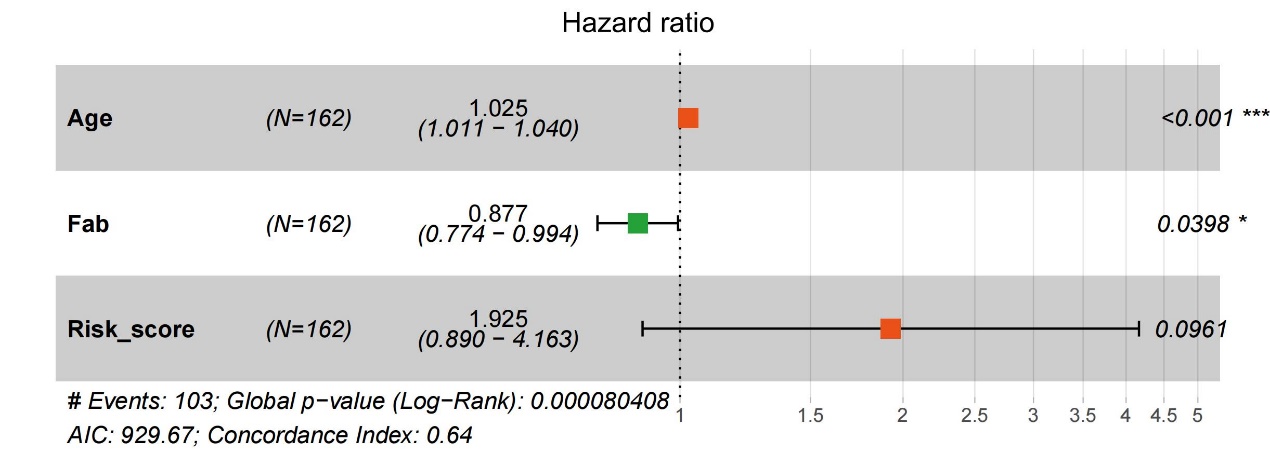


**Supplementary Figure S15.** Univariate Cox regression analysis of the risk score and clinical parameters (GSE12417, GPL96)


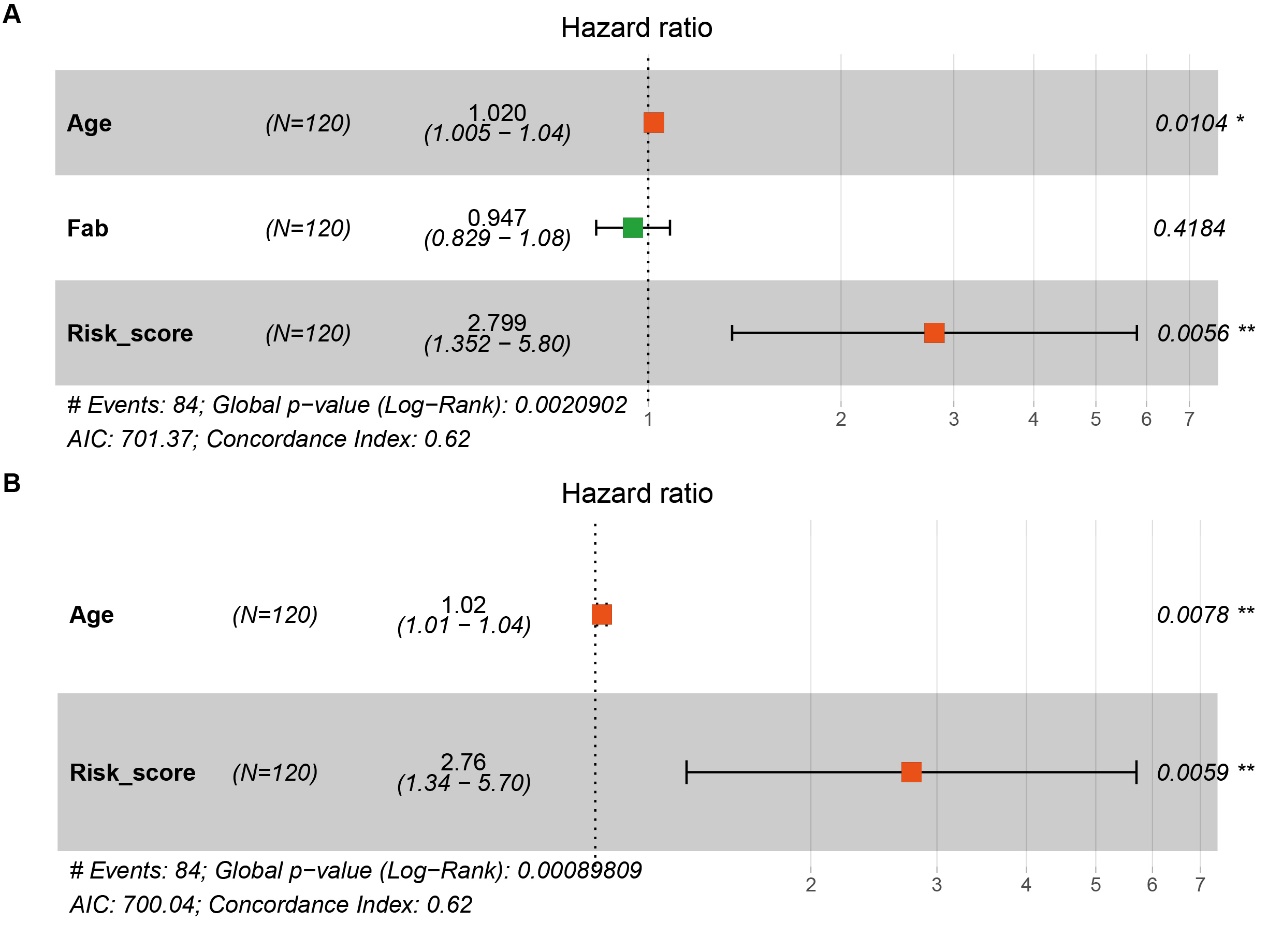


**Supplementary Figure S16.** Cox regression analysis of the risk score and clinical parameters (GSE37642, GPL570)

(A) Univariate Cox regression analysis (B) Multivariate Cox regression analysis

*p < 0.05; **p < 0.01; ***p < 0.001; ns, not significant.


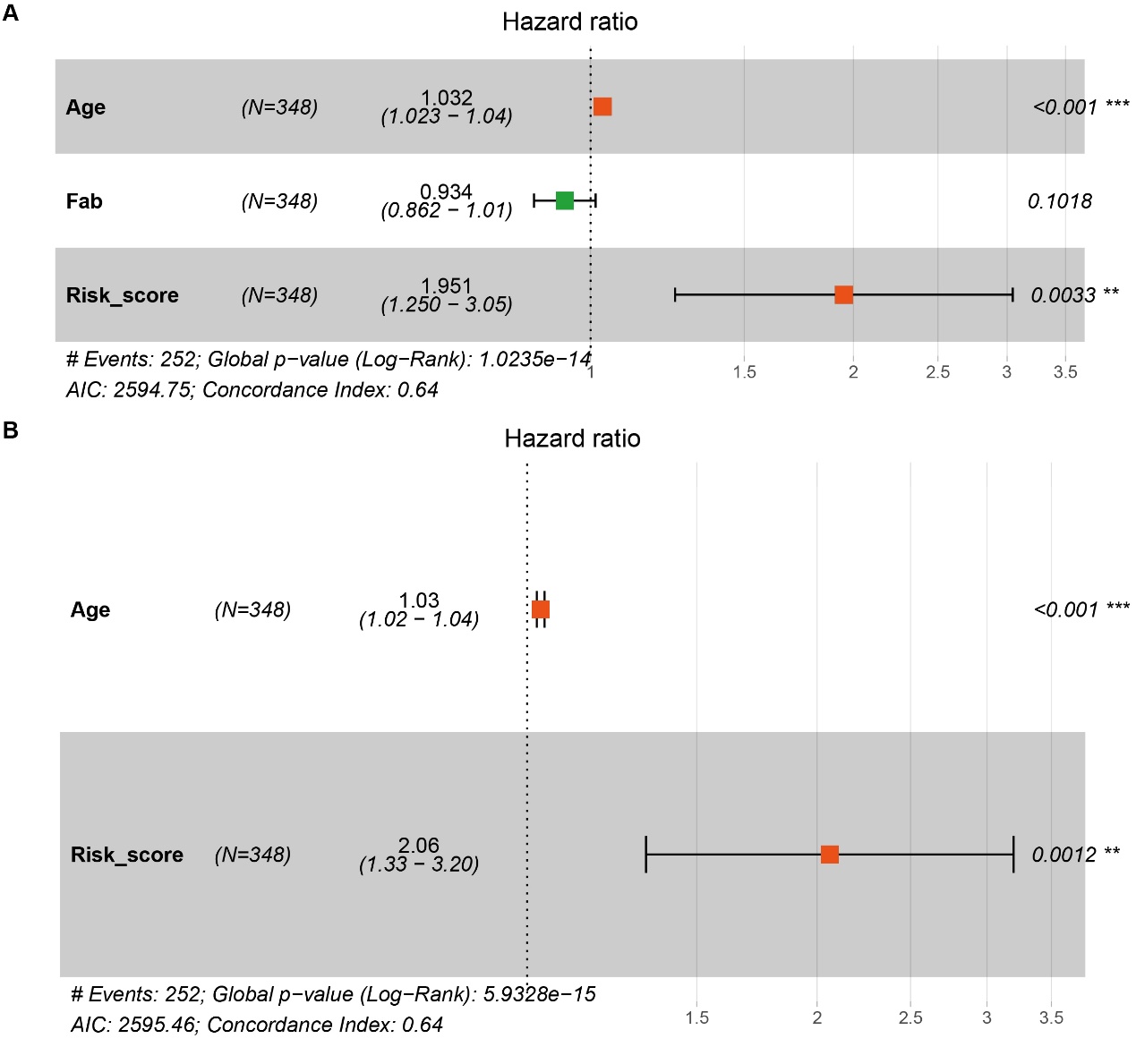


**Supplementary Figure S17.** Cox regression analysis of the risk score and clinical parameters (GSE37642, GPL96)

(A) Univariate Cox regression analysis (B) Multivariate Cox regression analysis

*p < 0.05; **p < 0.01; ***p < 0.001; ns, not significant.


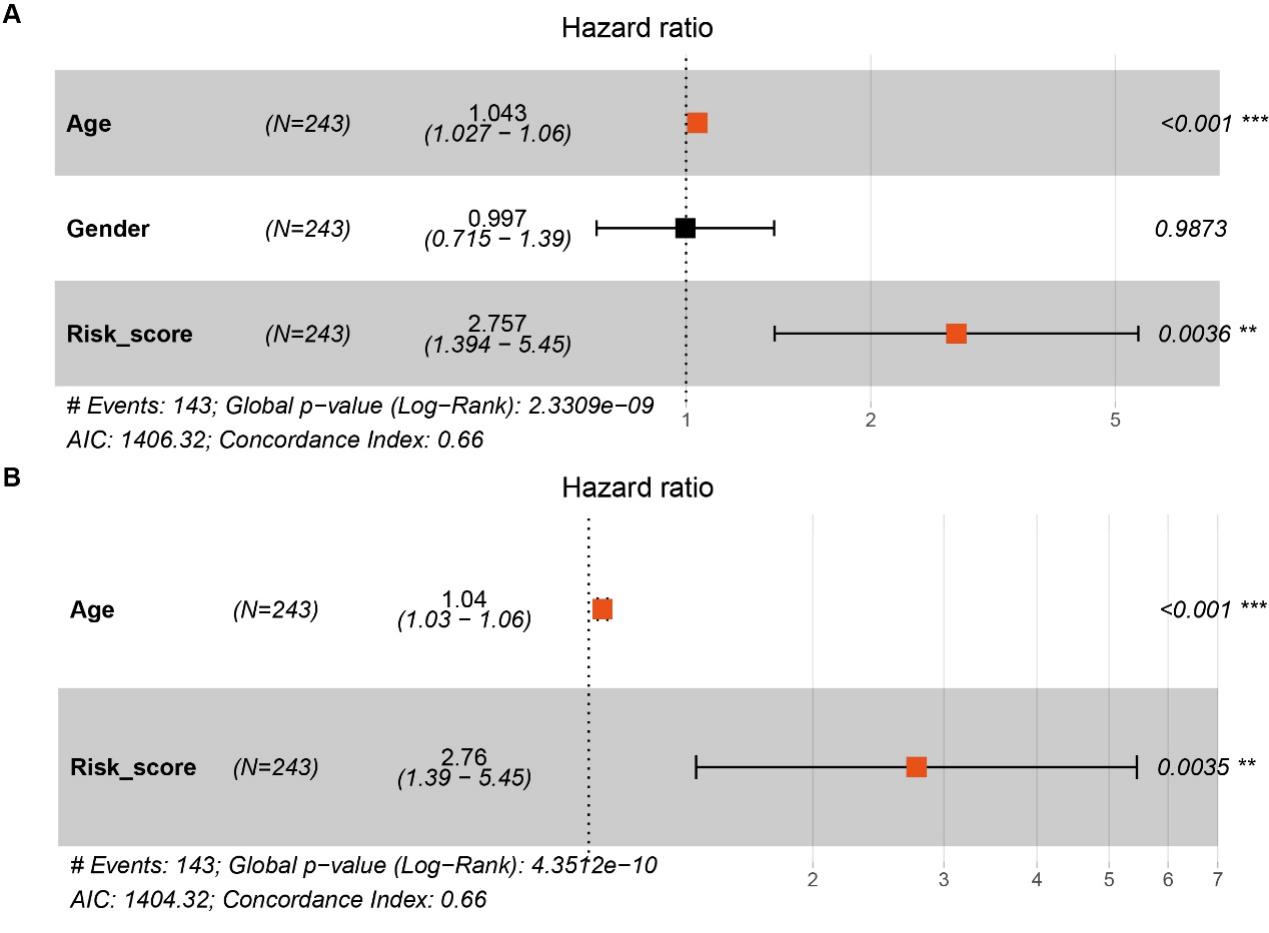


**Supplementary Figure S18.** Cox regression analysis of the risk score and clinical parameters (GSE106291, GPL18460)

(A) Univariate Cox regression analysis (B) Multivariate Cox regression analysis

*p < 0.05; **p < 0.01; ***p < 0.001; ns, not significant.


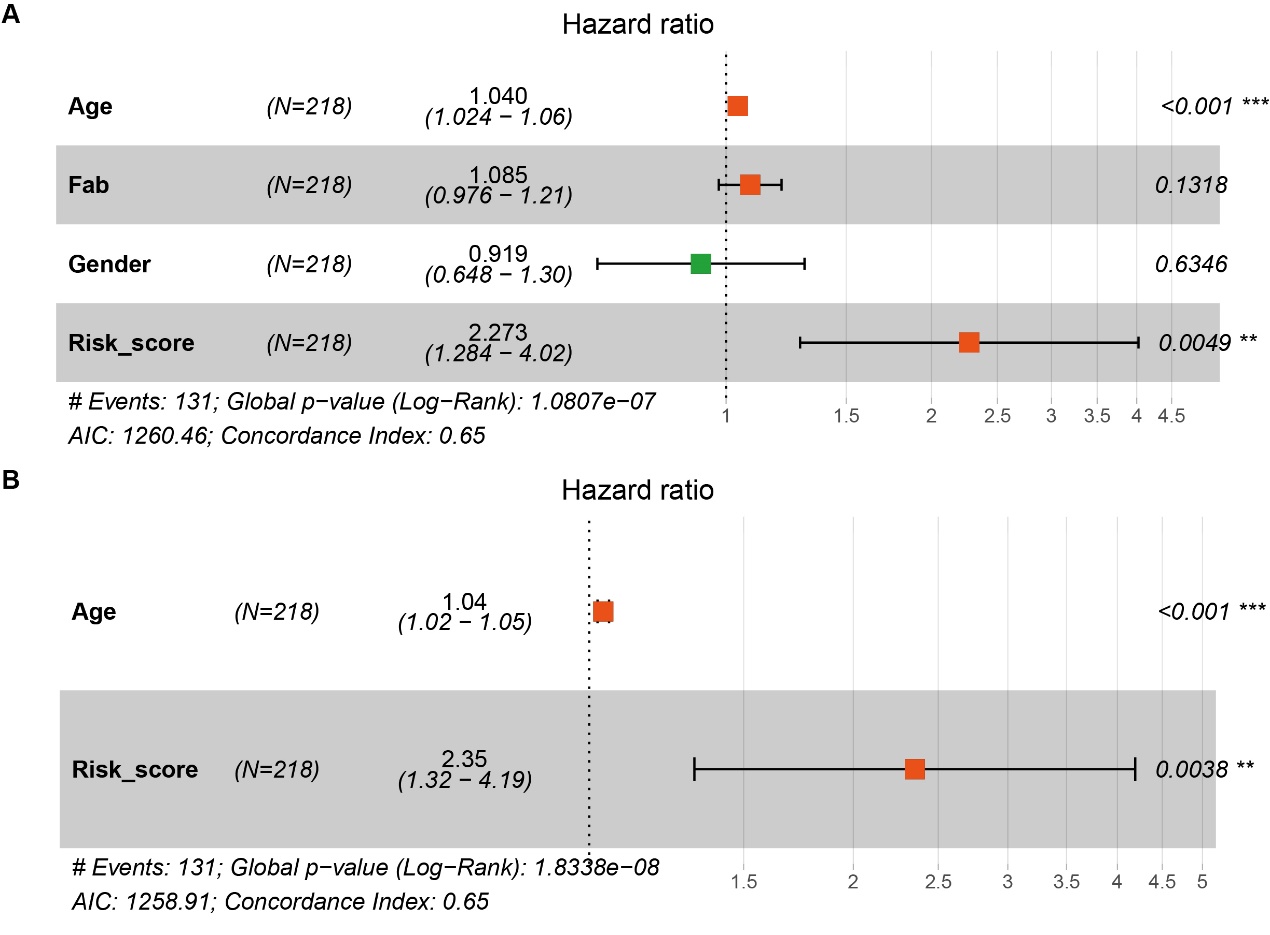
**Supplementary Figure S19.** Cox regression analysis of the risk score and clinical parameters (GSE146173, GPL18460)

(A) Univariate Cox regression analysis (B) Multivariate Cox regression analysis

*p < 0.05; **p < 0.01; ***p < 0.001; ns, not significant.


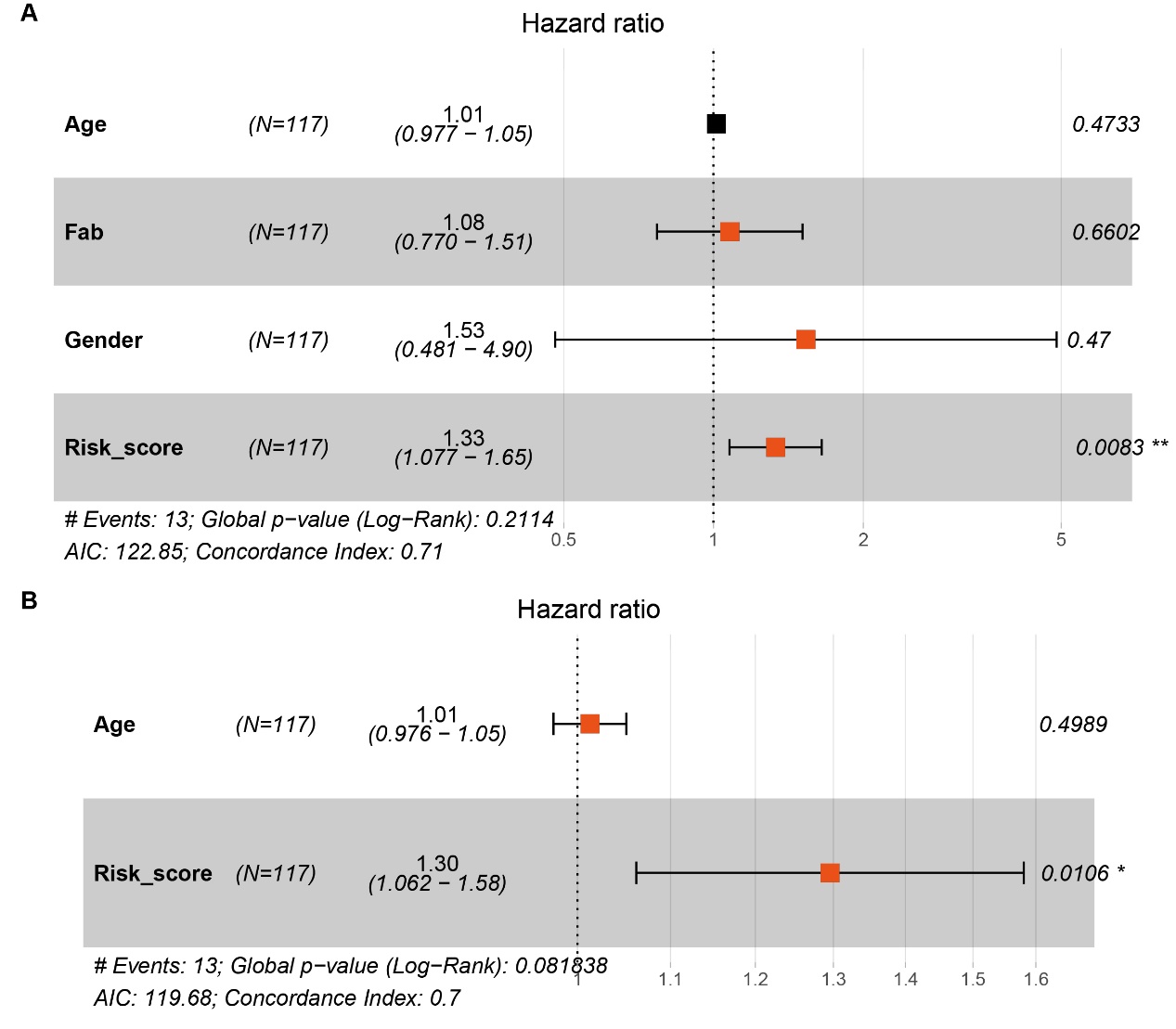


**Supplementary Figure S20.** Cox regression analysis of the risk score and clinical parameters (Our cohort).

(A) Univariate Cox regression analysis (B) Multivariate Cox regression analysis

*p < 0.05; **p < 0.01; ***p < 0.001; ns, not significant.


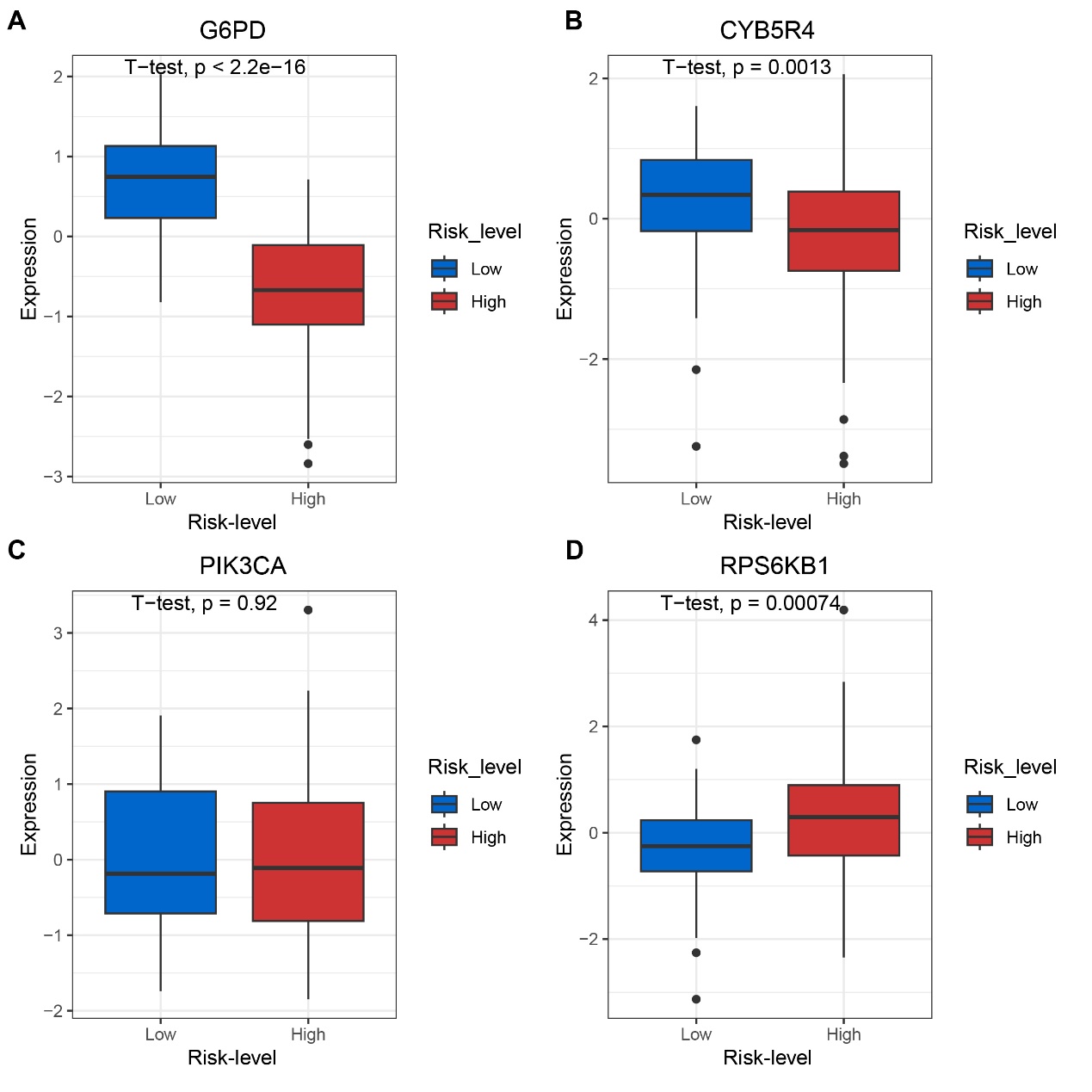


**Supplementary Figure S21.** mRNA expression levels of the 4 CARGs in high and low-risk groups (GSE12417, GPL96).


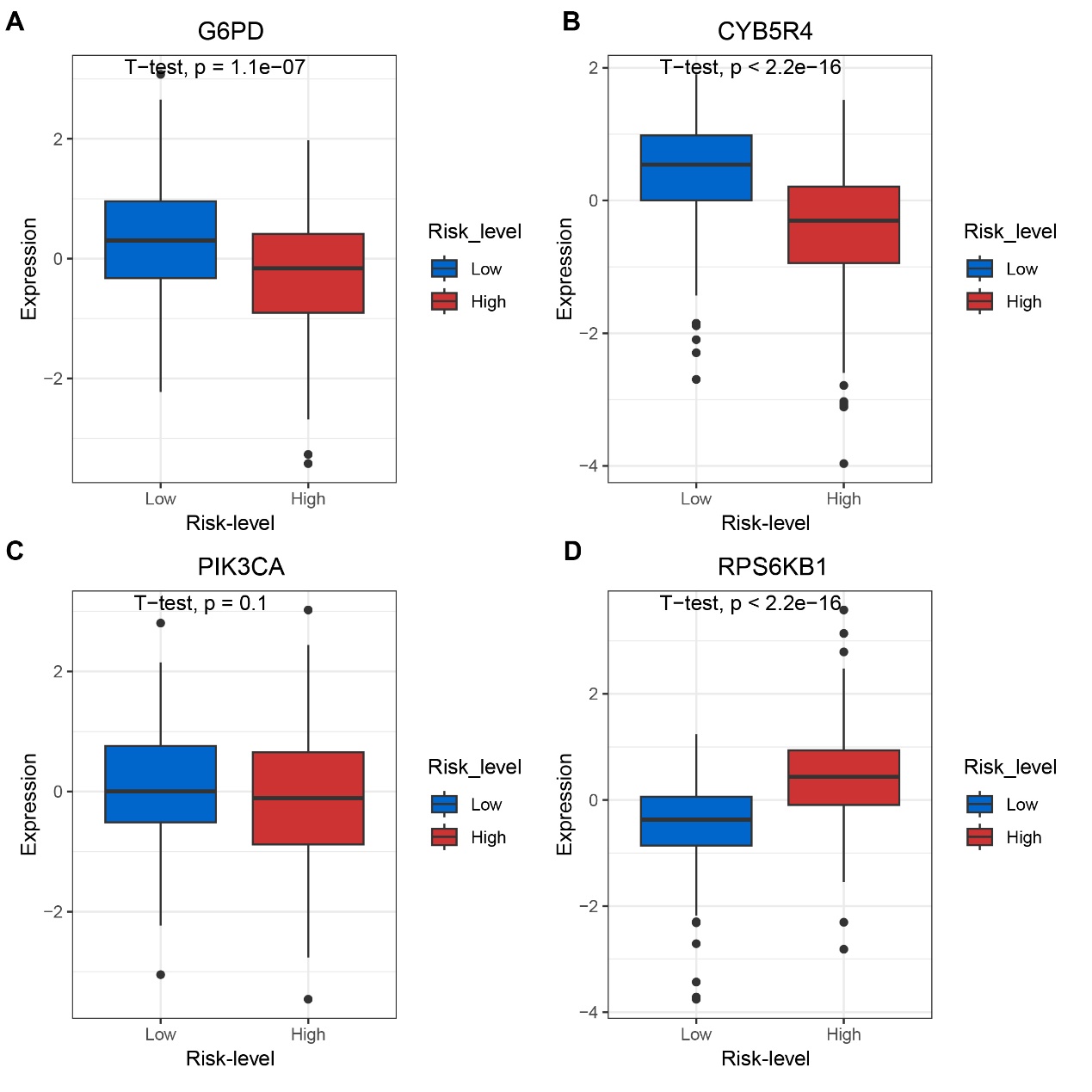


**Supplementary Figure S22.** mRNA expression levels of the 4 CARGs in high and low-risk groups (GSE37642, GPL570).


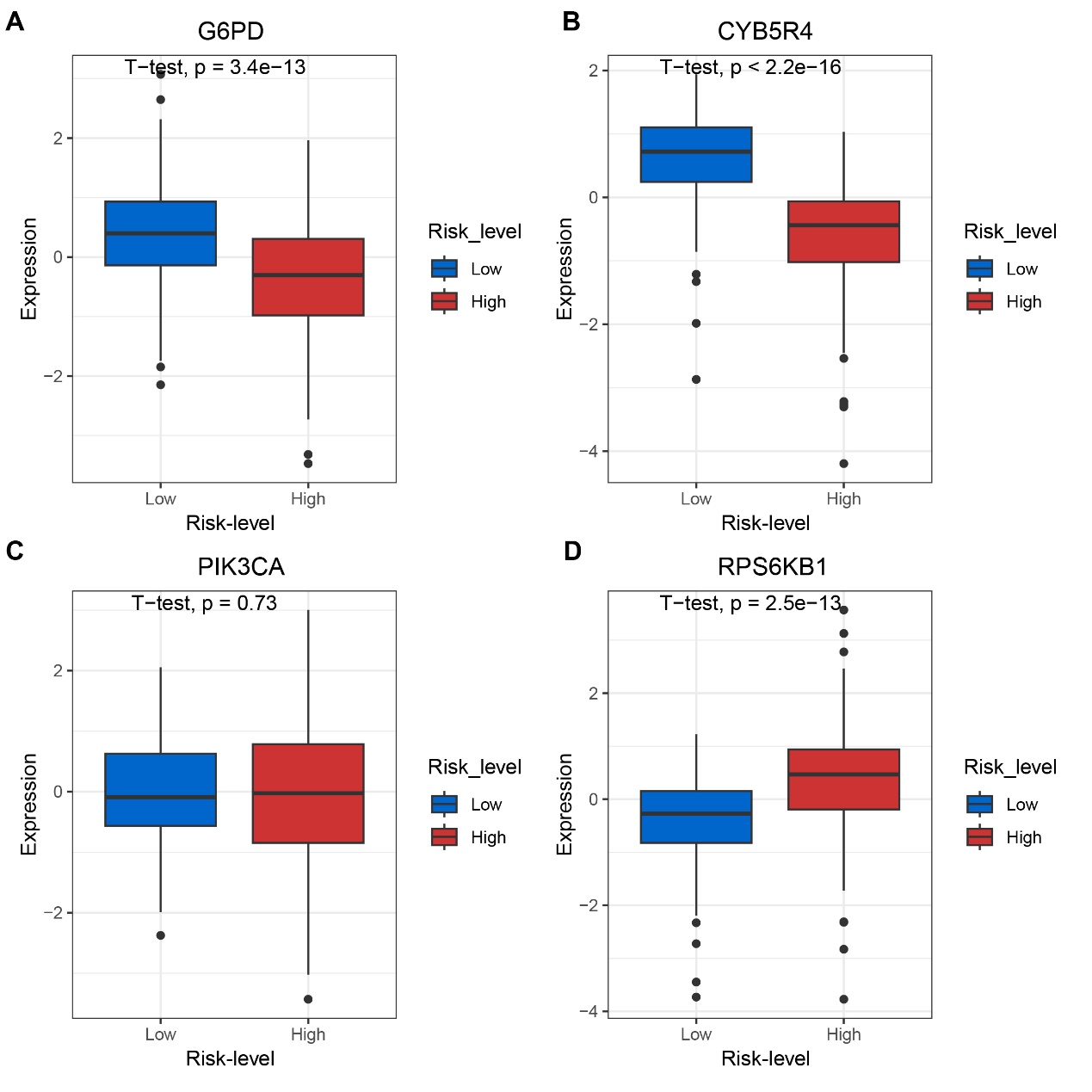


**Supplementary Figure S23.** mRNA expression levels of the 4 CARGs in high and low-risk groups (GSE37642, GPL96).


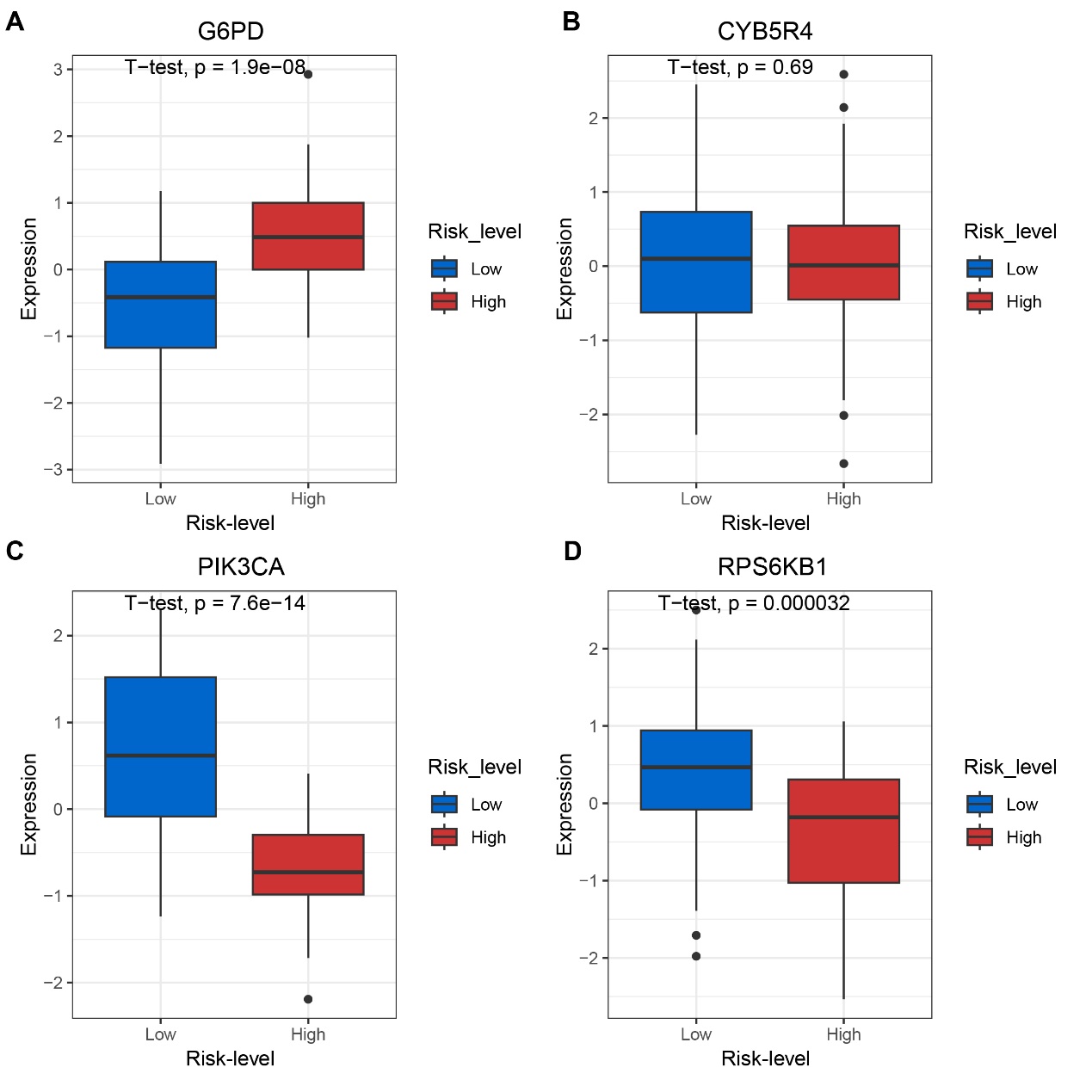


**Supplementary Figure S24.** mRNA expression levels of the 4 CARGs in high and low-risk groups (GSE71014, GPL10558).


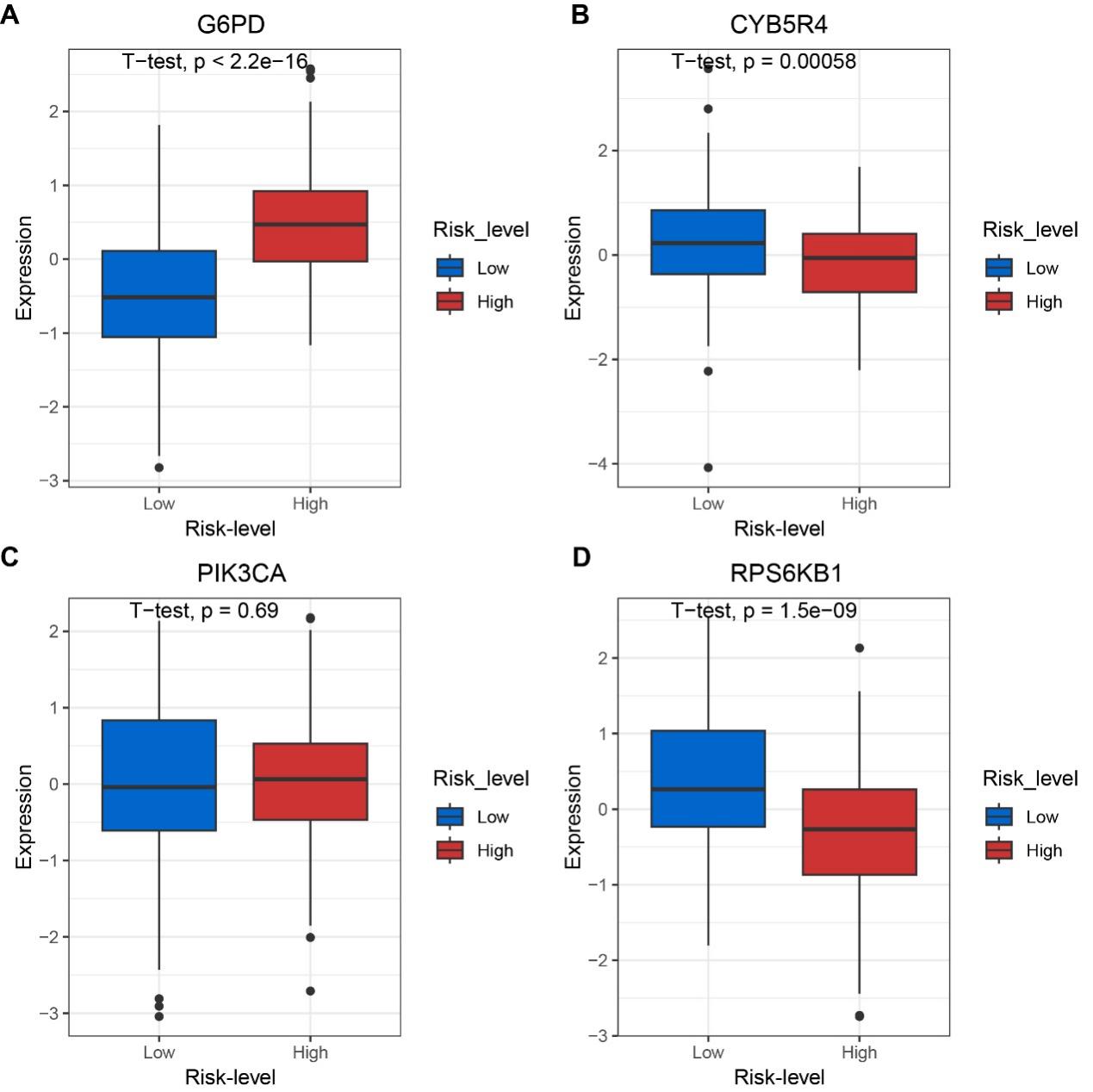


**Supplementary Figure S25.** mRNA expression levels of the 4 CARGs in high and low-risk groups (GSE106291, GPL18460).


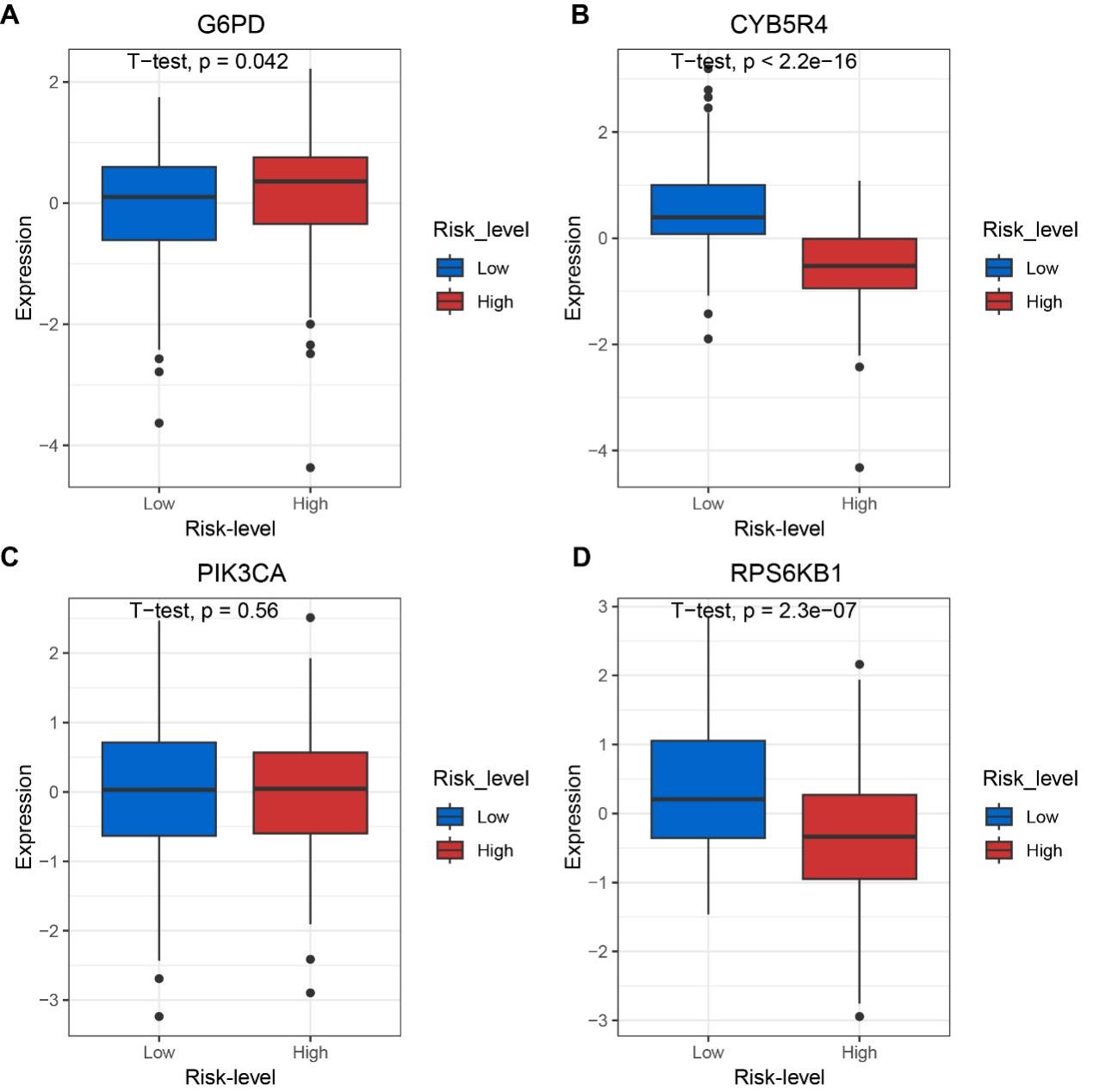


**Supplementary Figure S26.** mRNA expression levels of the 4 CARGs in high and low-risk groups (GSE146173, GPL18460).


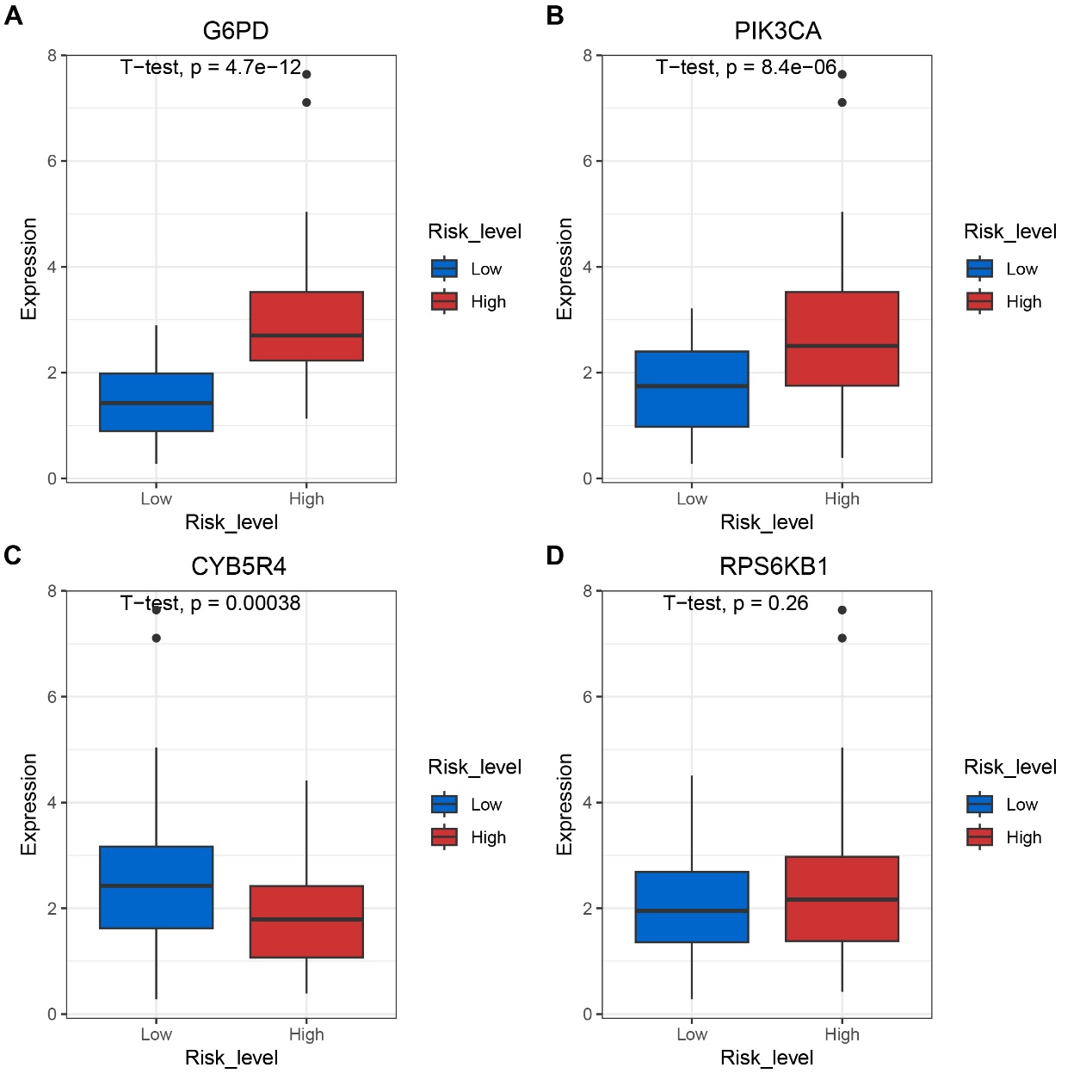


**Supplementary Figure S27.** mRNA expression levels of the 4 CARGs in high and low-risk groups (Our cohort).

**
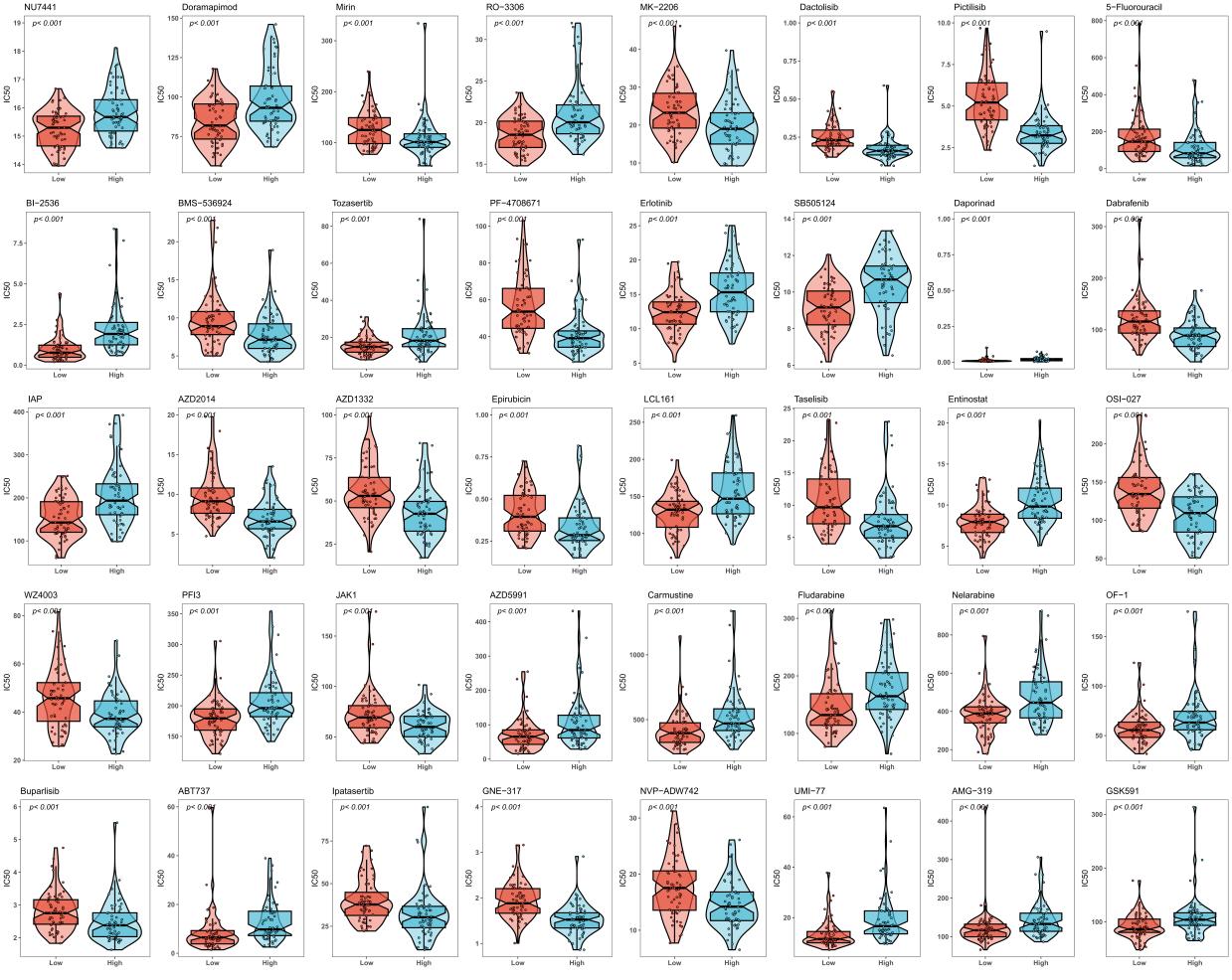
**

**Supplementary Figure S28.** Potential clinical chemotherapeutic response

IC50 indicates the half maximal inhibitory concentration.
